# Whole genome sequence analysis of rice genotypes with contrasting response to salinity stress

**DOI:** 10.1038/s41598-020-78256-8

**Published:** 2020-12-04

**Authors:** Prasanta K. Subudhi, Rama Shankar, Mukesh Jain

**Affiliations:** 1 School of Plant, Environmental, and Soil Sciences, Louisiana State University Agricultural Center, Baton Rouge, LA 70803 USA; 2 School of Computational and Integrative Sciences, Jawaharlal Nehru University, New Delhi, 110067 India; 3 Present Address: Department of Pediatrics and Human Development, Michigan State University, Grand Rapids, 49503 USA

**Keywords:** Genetics, Plant genetics

## Abstract

Salinity is a major abiotic constraint for rice farming. Abundant natural variability exists in rice germplasm for salt tolerance traits. Since few studies focused on the genome level variation in rice genotypes with contrasting response to salt stress, genomic resequencing in diverse genetic materials is needed to elucidate the molecular basis of salt tolerance mechanisms. The whole genome sequences of two salt tolerant (Pokkali and Nona Bokra) and three salt sensitive (Bengal, Cocodrie, and IR64) rice genotypes were analyzed. A total of 413 million reads were generated with a mean genome coverage of 93% and mean sequencing depth of 18X. Analysis of the DNA polymorphisms revealed that 2347 nonsynonymous SNPs and 51 frameshift mutations could differentiate the salt tolerant from the salt sensitive genotypes. The integration of genome-wide polymorphism information with the QTL mapping and expression profiling data led to identification of 396 differentially expressed genes with large effect variants in the coding regions. These genes were involved in multiple salt tolerance mechanisms, such as ion transport, oxidative stress tolerance, signal transduction, and transcriptional regulation. The genome-wide DNA polymorphisms and the promising candidate genes identified in this study represent a valuable resource for molecular breeding of salt tolerant rice varieties.

## Introduction

Salinity is a major environmental constraint that threatens world food security. It affects crop growth, development, and productivity due to reduced water uptake and increased concentration of salts^1^. Soil salinization is increasing at an alarming rate with 1.5 million hectares of land becoming unsuitable for agriculture each year and 50% of the cultivable land is predicted to be unsuitable for farming by 2050^2^. Salinity will continue to be a major constraint for crop production due to climate change and poor irrigation practices. Therefore, enhancing adaptation of major crop plants under saline condition and development of improved irrigation management practices are logical and pragmatic approaches for increasing global food production.

Rice (*Oryza sativa* L.) is a food staple for more than 50% of the world population. Since it is highly sensitive to salt stress, development of rice varieties that maintain high yield under saline environment is needed to enhance rice productivity. Abundant natural variability for salt tolerance existing in rice germplasm have been exploited in rice breeding programs with limited success^3,4^. Genetic engineering approaches using many genes associated with signaling pathways, ion transport, oxidative stress tolerance, and osmolyte accumulation have not led to commercialization of any salt tolerant rice variety. The lack of progress in this direction is mainly due to involvement of multiple adaptive mechanisms controlled by myriad of genes. Identification of genes and their superior variants would be helpful for a comprehensive understanding of salt tolerance mechanisms as well as for rice breeding activities through marker-assisted selection^5^.

Due to increased speed and reduced cost of sequencing, next generation sequencing technologies have accelerated development and adoption of novel strategies for crop improvement^6^. The available high-quality reference genome allows quick and precise alignment and mapping of sequences generated by next generation sequencing technologies resulting in identification of variants on a genome scale^7^. In general, discovery of single nucleotide polymorphisms (SNPs) and insertions/deletions (InDels) spread over the whole genome has proven to be useful for high throughput genotyping, genome mapping, population genetics studies, gene cloning, and marker-assisted breeding^7^. Since the DNA sequence variants particularly in the coding and regulatory regions impact gene expression, discovery of genome-wide variants provides an opportunity to elucidate the molecular basis of phenotypic differences.

The whole genome resequencing of rice germplasm has generated valuable genomic resources to accelerate both genetic analysis and molecular breeding of agronomically important traits^8–10^. A most significant undertaking in this regard involved sequencing of 3,010 diverse rice genotypes which revealed numerous novel protein coding genes and provided a comprehensive analysis of genetic diversity, population structure, and domestication process^11^. These genomic resources have been exploited for developing markers for genome-wide association studies of agronomically important traits^12^ and for resolving the origin of cultivated rice^13^.

The molecular basis of response to abiotic stresses such as drought and salinity has been investigated by whole genome resequencing^14^. The analysis of whole genome and transcriptome of a salt tolerant *indica* genotype SR86 revealed several differentially expressed genes and their high impact variants under salt stress^15^. Since very few studies have analyzed the genome level variation in salt sensitive and salt tolerant genotypes^14–16^, genomic resequencing in more diverse genetic materials is needed not only to provide insights into the molecular basis of salt tolerance, but also to generate genomic resources for rice improvement. In this study, we used three salt sensitive and two salt tolerant rice genotypes for resequencing. Compared to earlier studies^14,15^, we contrasted the salt tolerant genotypes with the salt sensitive genotypes at the whole genome level and identified genome-wide DNA polymorphisms. Through integration of the DNA polymorphism data with the results from our transcriptomics^16^ and quantitative trait loci (QTL) studies^17–19^, the differentially expressed genes (DEGs) and their variants present in the salt tolerance QTL regions were identified for validation as well as for use in molecular breeding to improve salt tolerance in the future.

## Results

### Genome-wide discovery of DNA polymorphisms in pair-wise analysis

Whole genome resequencing of five genotypes resulted in a total of 413 million reads (Table 1). Ninety-eight percent of the filtered reads were mapped on the reference genome. The mean genome coverage was 93% with highest in Bengal (97%) and lowest in IR64 (87%). The sequencing depth ranged from 12-fold in Cocodrie to 26-fold in IR64. The genome sequence of each of the two salt tolerant genotypes was compared with each of the three salt sensitive genotypes for discovery of SNPs and InDels. Together for all six combinations (IR64/Pokkali, IR64/Nona Bokra, Bengal/Pokkali, Bengal/Nona Bokra, Cocodrie/Pokkali, and Cocodrie/Nona Bokra) (Supplementary Fig. S1), largest number of SNPs and InDels were identified in chromosome 1, whereas chromosome 9 harbored the least. The frequency of SNPs and InDels was highest in chromosomes 6 and 7 and lowest in chromosome 10.

**Table 1:** Summary of sequence data and mapping statistics of the rice genotypes used in this study.

	Total raw reads	Total filtered reads	Sequencing depth (fold)	Total mapped reads	Genome coverage (%)	Uniquely mapped reads
Pokkali	68,001,168	63,188,954 (92.9%)	14.7	62,376,939 (98.7%)	92.7	45,061,913 (71.3%)
Nona Bokra	65,901,336	60,705,534 (92.1%)	14.1	59,995,162 (98.8%)	92.2	43,889,764 (72.3%)
Cocodrie	53,807,980	49,931,256 (97.5%)	11.6	49,478,104 (99.1%)	95.1	39,103,676 (78.3%)
Bengal	104,386,506	98,771,468 (94.6%)	23.0	98,132,017 (99.4%)	96.6	80,529,369 (81.5%)
IR64	121,235,526	113,048,322 (93.3%)	26.3	109,233,476 (96.6%)	86.7	79,987,053 (70.8%)

With regards to the individual pair-wise comparisons, the number of variants was highest in both Bengal/Pokkali and Bengal/Nona Bokra, but lowest in IR64/Pokkali and IR64/Nona Bokra (Supplementary Fig. S2). There were 1.3 million SNPs in each combination involving Bengal, whereas 105,027 and 109,626 InDels were identified in Bengal/Pokkali and Bengal/Nona Bokra combinations, respectively (Supplementary Tables S1 and S2). On the other hand, the number of SNPs was 536,863 and 549,297 and the number of InDels was 43,604 and 46,482 in IR64/Pokkali and IR64/Nona Bokra, respectively. The number of SNPs and InDels in combinations involving Cocodrie was in between the combinations involving Bengal and IR64. The frequency of SNPs and InDels per 100 kb was highest in combinations involving Bengal followed by Cocodrie and IR64.

The distribution and frequency of these DNA polymorphisms were uneven across the rice chromosomes in all six combinations (Supplementary Tables S1 and S2). The number of InDels was proportionate to the chromosome length but not for SNPs. For example, the largest number of SNPs was observed on the longest chromosome 1 in IR64/Pokkali, Bengal/Nona Bokra, Cocodrie/Pokkali and Cocodrie/Nona Bokra. The frequency of SNPs and InDels was not the lowest for the smallest chromosome. The frequency of SNPs was highest in chromosomes 12, 8, 9, 9, 6, and 7 in IR64/Pokkali, IR64/Nona Bokra, Bengal/Pokkali, Bengal/Nona Bokra, Cocodrie/Pokkali, and Cocodrie/Nona Bokra, respectively.

### Analysis and annotation of SNPs and InDels

Overall, the number of SNPs with transitions (A/G and C/T) was five-fold higher than SNPs with transversions (A/C, A/T, G/C, and G/T) (Supplementary Fig. S3A). Both A/G and C/T transitions were observed in equal number. The frequency of A/T, A/C, and G/T was similar but higher than G/C among the transversions. The transition/transversion ratio was 2.4. The length distribution revealed a range of 1 bp to 31 bp for deletions and 1 bp to 46 bp for insertions (Supplementary Fig. S3B).

Nearly 75% of SNPs and InDels were in the intergenic regions and the rest in the genic regions (Supplementary Fig. S4A). A large percentage of these polymorphisms were in promoter and 1 kb downstream regions. Most SNPs and InDels were present in the intronic regions (Supplementary Fig. S4B). The coding sequence (CDS) regions harbored a significantly higher percentage of SNPs compared with InDels. The percentage of SNPs and InDels in the 5′ untranslated regions (UTR) was the least. All SNPs identified in six combinations located within the CDS regions were classified into two categories, synonymous and nonsynonymous SNPs. There were more nonsynonymous SNPs than the synonymous SNPs. Of the 170,104 SNPs identified in the CDS regions, 56% were nonsynonymous. Among InDels, 2800 frameshift mutations were detected in the CDS regions.

The SNPs and InDels were categorized into mainly four types based on their functional annotation: high impact (affecting splice site, stop, and start codons), moderate impact (nonsynonymous), low impact (synonymous coding, alternate start/stop, start gained), and modifier (upstream, downstream, intergenic, and UTRs). The share of modifier SNPs was the highest among all SNPs (93.5%) followed by moderate (3.5%), low (2.7%), and high impact SNPs (0.2%) (Supplementary Table S3). On the other hand, 98.3% of InDels were modifiers, but the number of high impact InDels were more (1.1%) compared to moderate (0.2%) and low impact InDels (0.4%). The high and moderate impact SNPs affecting the amino acids were assigned into missense, nonsense, and silent categories, which constituted 58%, 2.7% and 39.7%, respectively (Supplementary Table S4). In addition, only a small number of high impact SNPs and InDels affecting the transcript splice site (splice acceptor and splice donor), stop codon (stop site gain and stop site lost) and start codon lost were identified.

### Genes carrying SNPs and InDels and their functional relevance

The genes carrying nonsynonymous/large effect SNPs/InDels were classified based on their functions using eukaryotic orthologous group (KOG) analysis (Supplementary Fig. S5). There was no difference in percentage of genes under each category in both Pokkali and Nona Bokra comparisons. The largest group included genes for general function followed by signal transduction mechanism, post translational modification, protein turnover, and chaperones. Few other highly enriched groups included genes involved in RNA processing function, energy production and conversion, cell cycle control, cell division, amino acid transport, carbohydrate transport, lipid transport, transcription, and translational activities.

Gene ontology (GO) analysis was performed using the three sets of genes identified in all six genotype pairs: (a) genes harboring the large effect/nonsynonymous SNPs and InDels, (b) genes harboring SNPs and InDels in the promoter regions, and (c) differentially expressed genes^16^ harboring large effect SNPs and/or nonsynonymous SNPs and InDels in the promoter regions. In case of the large effect variants containing genes, GO analysis revealed significant enrichment of genes involved in biological processes, such as cellular response to stress, ion transport, post-translational protein modification, phosphorylation and cellular carbohydrate, and lipid metabolic process, whereas the genes with protein binding activity, kinase activity, transferase activity, and hydrolase activity were abundant under the molecular function category (Supplementary Fig. S6). The analysis of polymorphisms in the promoter regions of genes revealed enrichment of similar categories of pathways but the genes involved in ATPase activity were significantly enriched (Supplementary Fig. S7). In case of DEGs harboring large effect SNPs and InDels, genes involved in the biological process such as response to chemical stimulus were enriched, whereas genes involved in DNA polymerase activity, kinase activity, phosphoprotein phosphatase activity were significantly abundant under the molecular function category (Supplementary Fig. S8).

### Analysis of DNA polymorphisms between salt tolerant and salt sensitive genotypes

**Figure 1:**
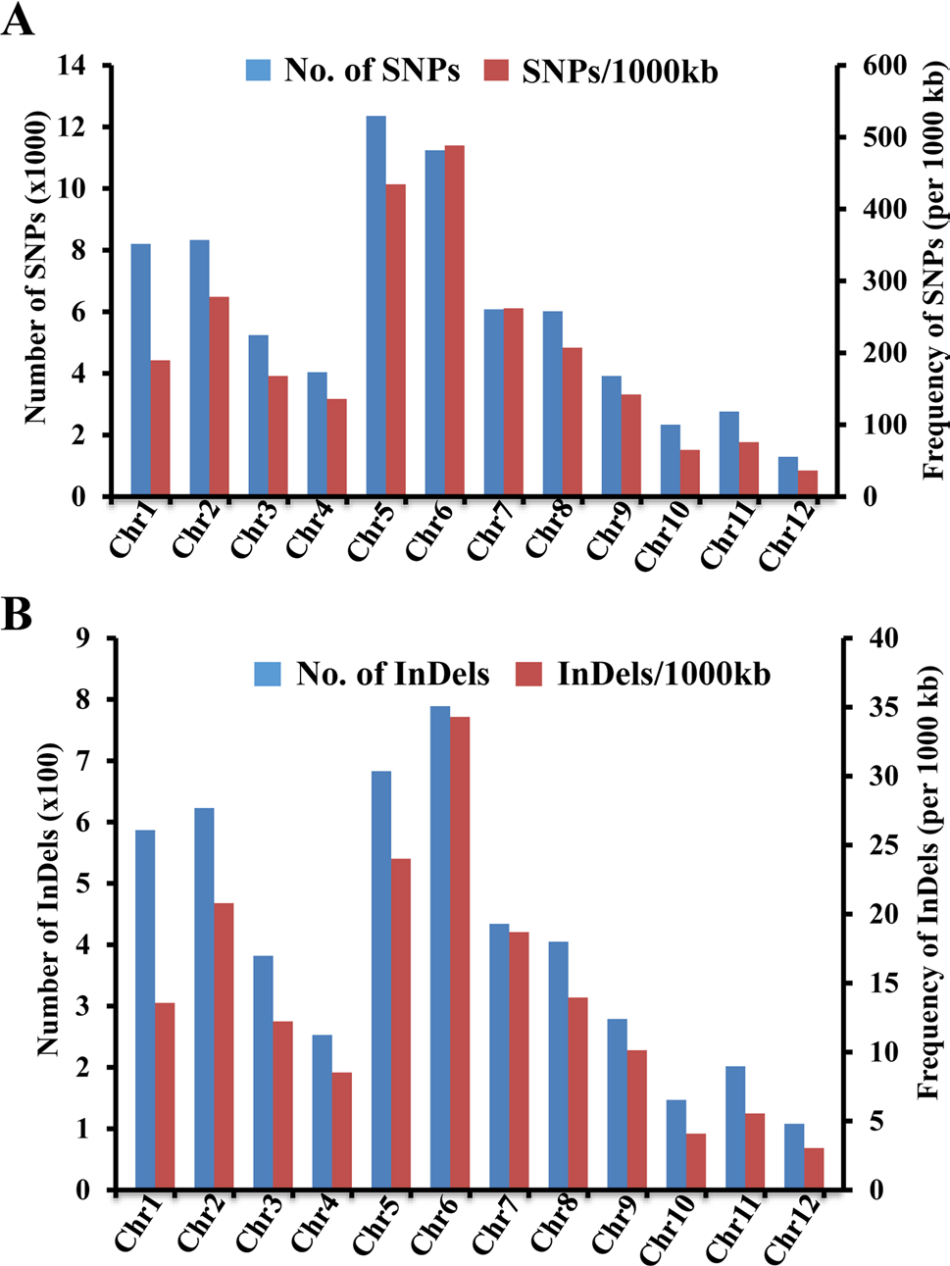
Distribution and frequency of SNPs and InDels on each rice chromosome. Number and frequency (per 1000 kb) of SNPs (**A**) and InDels (**B**) showing 100% allelic variations in salt sensitive and salt tolerant rice genotypes detected on each rice chromosome are shown in bar graphs.

**Figure 2:**
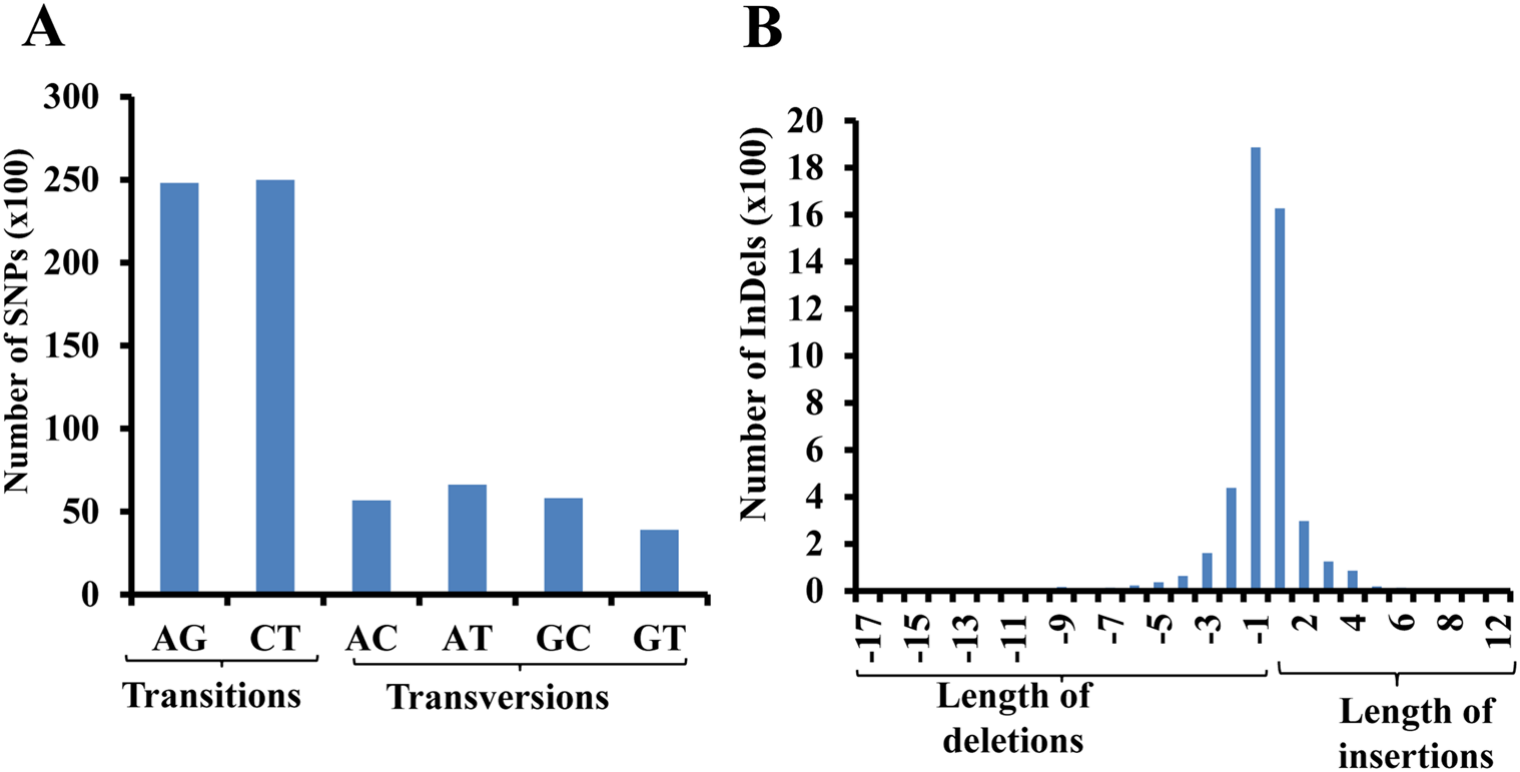
Frequency of substitution types in SNPs and length distribution of InDels. (**A**) Number of different substitution types in the identified SNPs showing 100% allelic variations in salt sensitive and salt tolerant rice genotypes. (**B**) Length distribution of InDels showing 100% allelic variations in salt sensitive and salt tolerant rice genotypes. Number of insertions and deletions (y-axis) of various lengths (x-axis, in bp) are shown in bar graphs.

The distribution and frequency of genome-wide DNA polymorphisms and their functional relevance between the salt tolerant and salt sensitive genotypes were examined to gain insights into the molecular basis of salt tolerance (Fig. 1). All polymorphic SNPs and InDels between these two groups were considered for analysis. The frequency and density of these polymorphisms were not proportional to the length of chromosomes which was quite different from the pattern seen in pairwise analysis (Supplementary Fig. S1). The number and density of SNPs and InDels per 100 kb was highest in chromosomes 5 and 6 and lowest in chromosomes 10 and 12. The type of nucleotide substitutions (transitions and transversions) followed the same trend as described before for the SNPs and InDels detected in all six pairs and the ratio of transition/transversion was slightly lower than the estimate made on all combinations (Fig. 2A). The length of insertions and deletions detected was up to 12 bp and 17 bp, respectively and most polymorphisms involved insertion or deletion of 1–2 bp (Fig. 2B).

**Figure 3:**
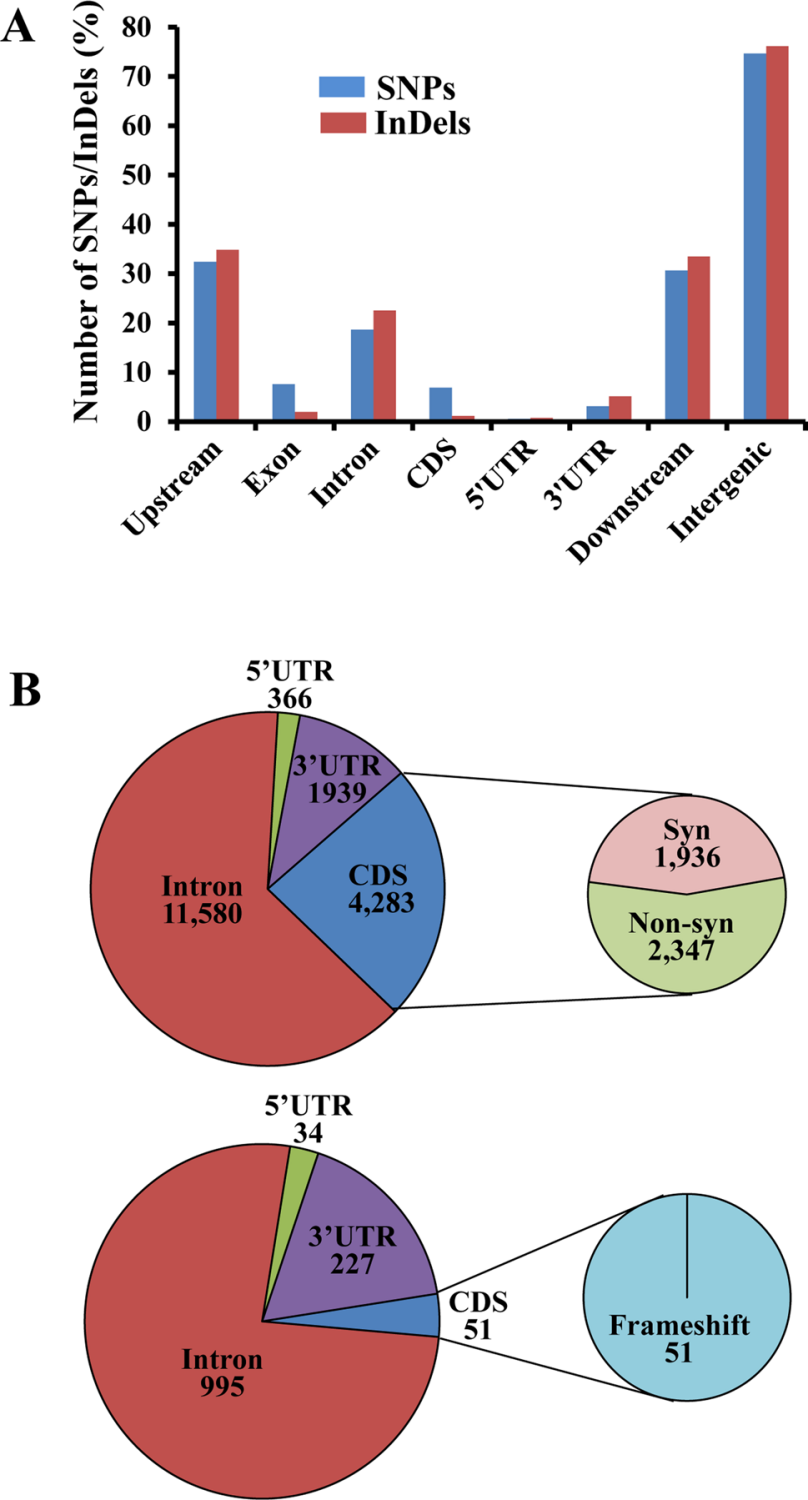
Annotation of SNPs and InDels showing 100% allelic variations in salt sensitive and salt tolerant rice genotypes. (**A**) Distribution of SNPs and InDels in different genomic regions. (**B**) Distribution of SNPs and InDels in different genic regions. The number of synonymous (syn) and nonsynonymous (non-syn) SNPs and number of frameshift mutations detected within the CDS regions were shown for SNPs (top) and InDels (bottom), respectively.

Analysis of annotation information from rice genome revealed that majority of DNA polymorphisms were in intergenic, intronic, upstream, and downstream regions of the genes (Fig. 3A). A total of 18,168 SNPs and 1307 InDels were located within the genes and 24% of these SNPs and 4% of InDels were present in the CDS regions (Fig. 3B). Majority of SNPs (64%) and InDels (76%) were located in the intronic locations. There were only 2347 nonsynonymous SNPs and 51 frameshift changes detected between salt tolerant and salt sensitive groups.

A total of 85 SNPs and 56 InDels with large effects were identified (Table 2). Most of these high impact SNPs disrupted the splice sites, start, and stop codons. Among these groups, stop gained type constituted 55% of total polymorphisms. The high impact InDels were largely due to frameshift mutations. Compared to this result, large number of large effect SNPs and InDels was observed in each of the six combination (Supplementary Table S5).

**Table 2:** Large-effect single-nucleotide polymorphisms (SNPs) and InDels detected between salt tolerant genotypes (Pokkali and Nona Bokra) and salt sensitive genotypes (Bengal, Cocodrie, and IR64) as groups.

Type of DNA polymorphism	SNPs	InDels
Splice site acceptor	14	0
Splice site donor	10	0
Start lost	4	0
Stop lost	8	5
Stop gained	49	0
Frameshift	0	51
Total	85	56

**Figure 4:**
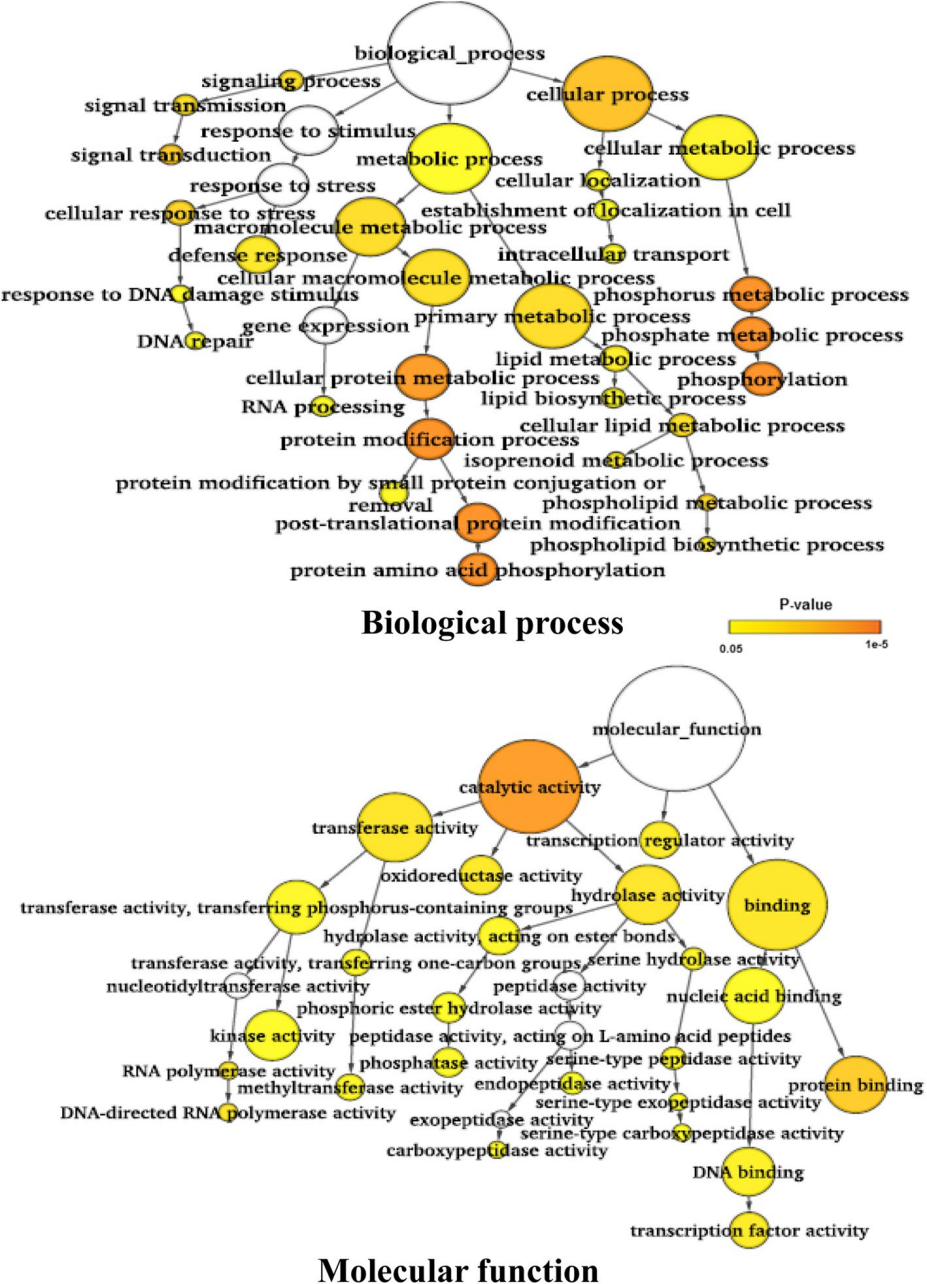
Gene ontology enrichment analysis for the genes harboring large effect/nonsynonymous SNPs and InDels. SNPs and InDels showing 100% allelic variations between salt sensitive and salt tolerant rice genotypes were used for analysis.

**Figure 5:**
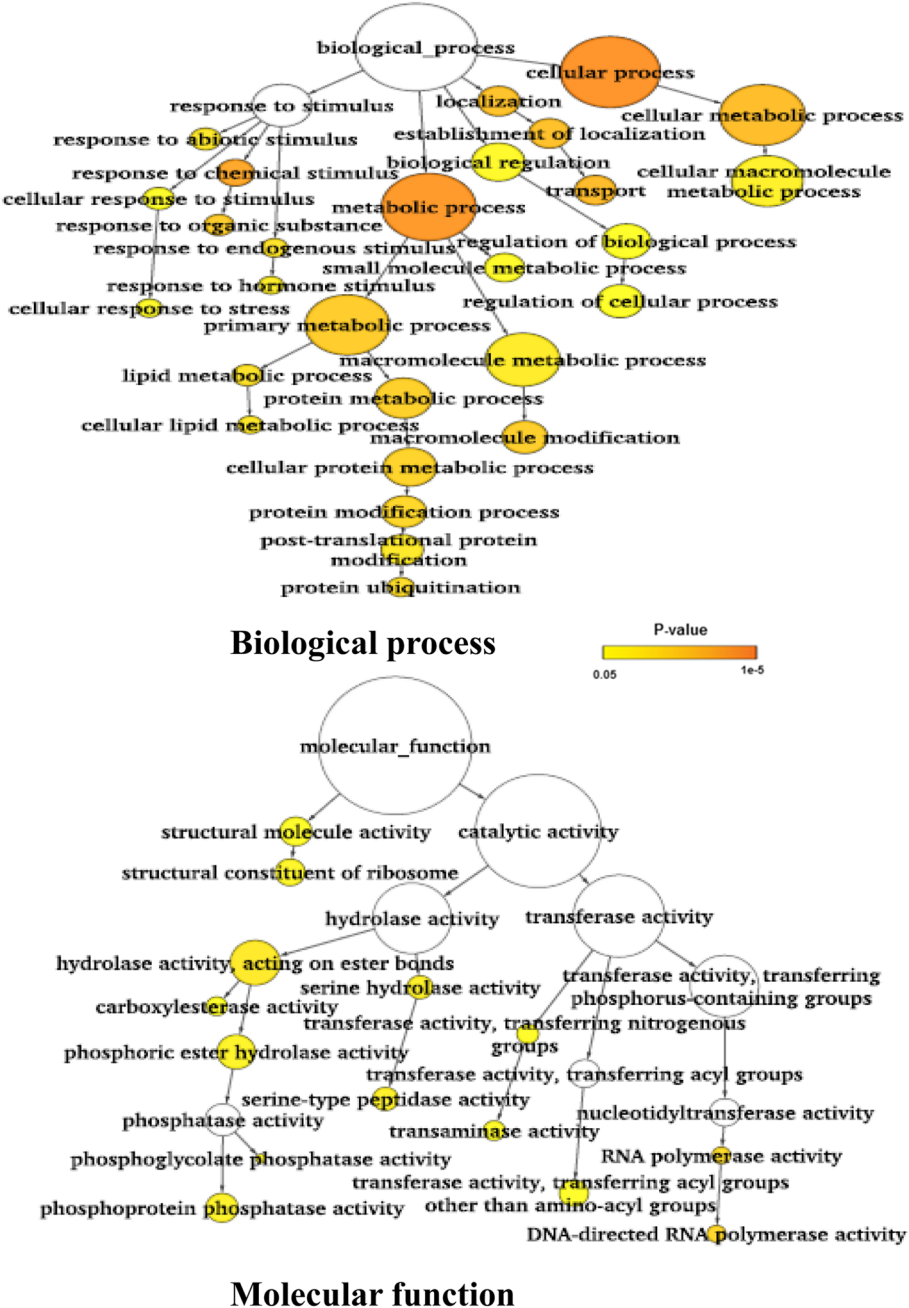
Gene ontology enrichment analysis for the genes harboring SNPs/InDels in promoter region. SNPs and InDels showing 100% allelic variations between salt sensitive and salt tolerant rice genotypes were used for analysis.

**Figure 6:**
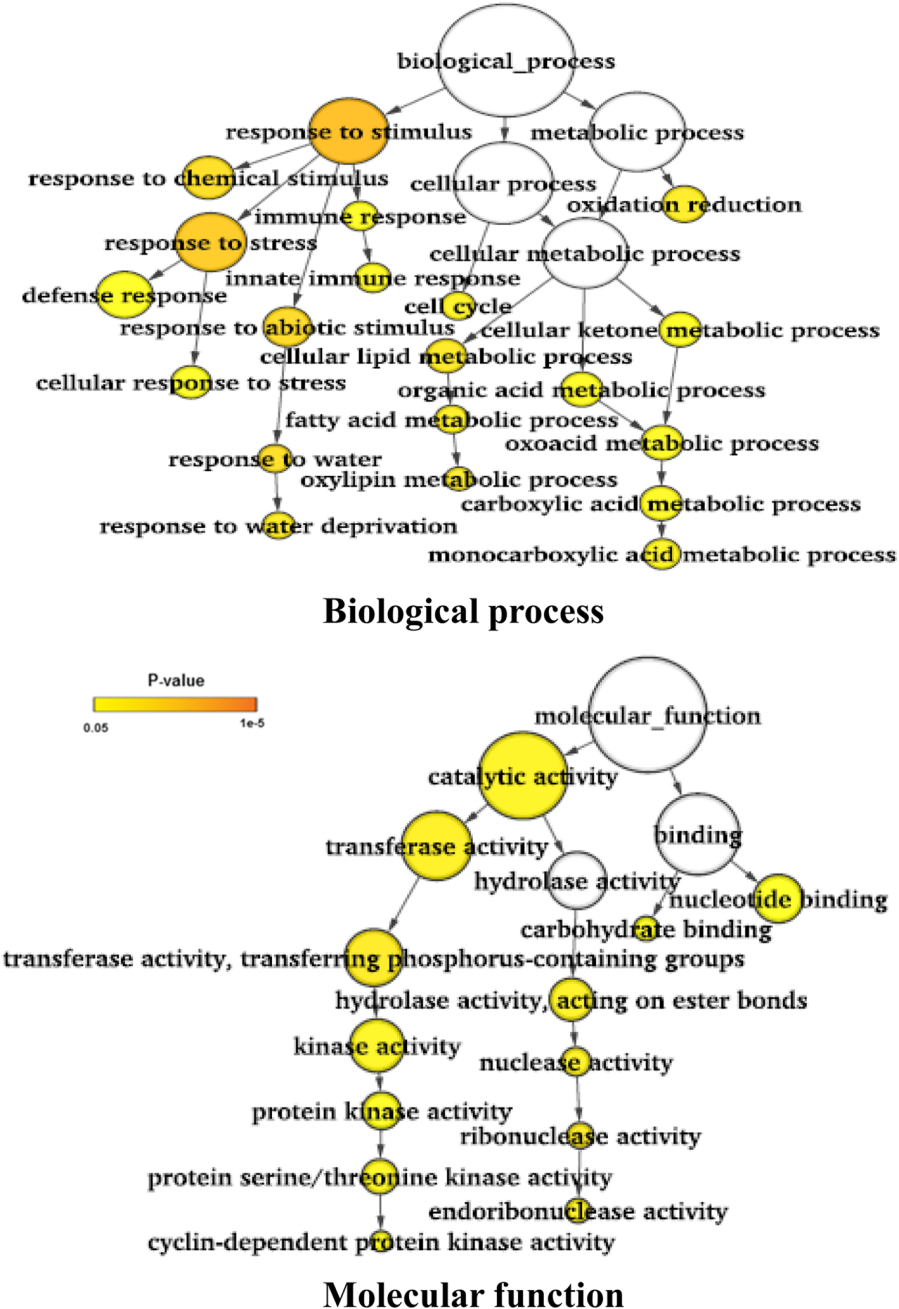
Gene ontology enrichment analysis for the differentially expressed genes (DEGs) harboring large effect SNPs/InDels and/or nonsyn SNPs and SNPs/InDels in the promoter regions. SNPs and InDels showing 100% allelic variations between salt sensitive and salt tolerant rice genotypes were used for analysis.

The gene ontology analysis using the genes carrying nonsynonymous SNPs and/or large effect DNA polymorphisms between the two groups revealed that genes involved in post-translational protein phosphorylation and protein binding genes were enriched (Fig. 4). But when the SNPs and InDels in the promoter regions were considered, the genes involved in post-translational protein ubiquitination and DNA-directed RNA polymerase activity were abundant (Fig. 5). But in case of the differentially expressed genes, genes involved in biological processes such as response to chemical stimulus and abiotic stimulus were enriched like an earlier study^16^ (Fig. 6).

### Associating salt tolerant QTLs to salt responsive genes

We selected 118 QTLs identified earlier in three mapping populations involving both Pokkali and Nona Bokra (Supplementary Table S6). These mapping populations were a RIL population from the cross Bengal x Pokkali^17^ and two introgression line populations from the crosses Jupiter x Nona Bokra^18^ and Cheniere x Nona Bokra^19^. Since a RNA-seq experiment conducted by our group identified DEGs between salt tolerant genotype Pokkali and salt sensitive IR64 in response to salt stress^16^, the expression data from this study was integrated with the DNA polymorphism data for the genes present in the salt tolerant QTL regions. This analysis resulted in identification of 1092 and 1511 DEGs with large effect SNPs/InDels in their coding and promoter regions, respectively (Supplementary Tables S7, S8).

**Figure 7:**
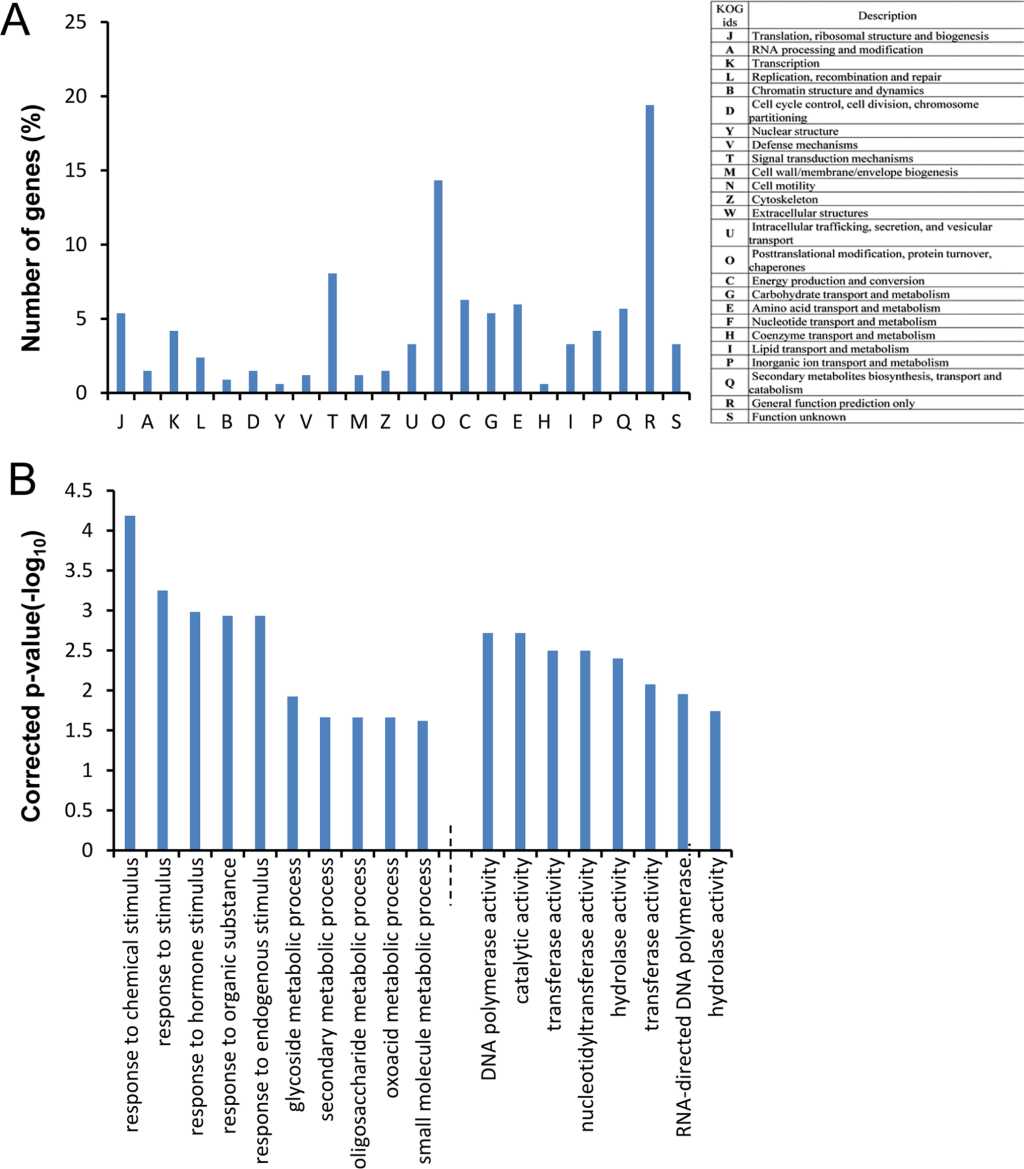
Functional categorization of differentially expressed genes harboring SNPs/InDels present in salinity related QTLs. (**A**) Distribution of genes in different eukaryotic orthologous groups (KOG). (**B**) Significantly enriched (Corrected P value ≤ 0.05) biological process (left) and molecular function (right) gene ontology terms are shown.

The eukaryotic orthologous groups analysis of DEGs in the QTL intervals (Fig. 7A) corroborated the earlier GO analysis (Figs. 5, 6). Fifteen percent of genes were involved in post-translational modification/protein turnover, and chaperones, whereas the signal transduction mechanisms accounted for 8% of the genes. Two other groups included genes involved in energy production and conversion (6%), and genes involved in amino acid transport and metabolism, secondary metabolite biosynthesis, transport and catabolism, and translation, ribosomal structure and biogenesis (5%). Genes associated with biological processes such as responses to chemicals, hormones, and organic substances were most abundant (Fig. 7B). On the other hand, the most enriched genes under the ‘molecular function’ were involved in DNA polymerase, catalytic, transferase, and hydrolase activity.

## Discussion

Salinity is a major climate-related risk for rice production worldwide. The discovery of DNA polymorphisms on a genome scale as well as salt stress related candidate genes are useful resources to facilitate genetic analysis and marker-assisted breeding activities for rice improvement. We focused on six pairs of comparisons involving two salt tolerant and three salt sensitive genotypes. There were differences in intraspecies comparisons as expected. For example, *indica/indica* comparison normally resulted in lower density of polymorphisms compared to *indica/japonica* comparison^10,20^. Higher number of polymorphisms observed in pairs involving Bengal compared to those involving Cocodrie could be due to genetic closeness of Cocodrie to *indica* genotypes. Even though both Bengal and Cocodrie belong to the *japonica* group, Bengal was genetically distinct from Cocodrie^21^. In all six pairs, the chromosomal regions with high and low density were identified for SNPs and InDels as previously reported^16,22,23^. Such localization of variants in several genomic regions could be due to hitch-hiking effect of many selected genes during the domestication process^24^.

Comparison of whole genome sequences between the salt tolerant and salt sensitive groups was done to identify the genes and their variants associated with salt tolerance (Fig. 1). Many of these variants differentiating both groups are expected to be related to salt tolerance. Since both salt tolerant genotypes were land races and all three salt sensitive genotypes were improved varieties, variants for genes involved in contrasting agronomic performance could be included. Compared to the pairwise analysis, there was a drastic reduction in number of SNPs and InDels between both groups. Subsequent annotation of these variants resulted in only 2347 nonsynonymous SNPs and 51 frameshift mutations (Fig. 3).

Integrating genome-wide polymorphism information to QTL mapping and transcriptomics data is increasingly used to identify candidate genes for the target traits^25,26^. In one study, QTL mapping coupled with RNA-seq analysis resulted in identification of 4 DEGs in the QTL regions for salt tolerance in rice^25^. But in this study, we first identified genome-wide DNA polymorphisms between two sets of genotypes with contrasting response to salinity stress. In the second step, we used the genomic coordinates of QTLs for several seedling salt tolerance traits from our previous studies involving the same two salt tolerant donors used in this study^17–19^ and identified the genes or their promoter regions carrying variants in the QTL confidence intervals. Finally, we determined the differentially expressed genes with large effect variants in the CDS and promoter regions using the gene expression data from an earlier study^16^ (Supplementary Tables S7 and S8).

The functional relevance of the DEGs present in the QTL intervals provides a better understanding of physiological and molecular mechanisms associated with adaptation in saline environment. There were 396 and 573 genes that carried variants in the CDS and promoter regions, respectively. Most abundant classes include genes involved in ion transport, oxidative stress tolerance, signal transduction, stress response, and transcriptional regulation (Supplementary Tables S7 and S8). Several transcriptomic studies reported involvement of genes associated with these salt tolerance mechanisms^15,27,28^. The congruence of salt tolerance QTLs with many protein kinase and leucine-rich repeat, zinc-finger, NB-ARC, and P450 domain containing genes was earlier reported^14^.

The DEGs involved in ion transport and homeostasis included potassium channel SKOR (LOC_Os06g14030) and chloride channel protein (LOC_Os04g55210), *OsHKT2;3*—Na^+^ transporter (LOC_Os01g34850), ABC transporter, ATP-binding protein (LOC_Os08g30740), AAA family ATPase, (LOC_Os02g19150), calcium-transporting ATPase, plasma membrane-type (LOC_Os12g04220), cation transport regulator-like protein 1 (LOC_Os02g26700), and sulfate transporters (LOC_Os01g41050, LOC_Os03g09970, LOC_Os03g09980). Reduced expression of *OsHKT2;4,* a high-affinity Na^+^/K^+^ transporter and SKOR in response to salt stress was previously reported^28^. There were three multi-antimicrobial extrusion (MATE) protein family genes (LOC_Os02g45380, LOC_Os01g31980, LOC_Os10g20350) with role in tolerance to abiotic stresses including salinity^29^.

The oxidative stress alleviating genes included several members of glutathione S-transferases, peroxidases, oxidoreductases, and HAD superfamily phosphatase. These genes were differentially expressed in rice under salt stress^15^. Several members of the cytochrome P450 family with role in growth, development, and stress tolerance^30^ were localized in the QTL intervals. The most abundant differentially expressed transcription factors were from MYB, AP2, NB-ARC, Zinc Finger, and Zinc knuckle families which were upregulated in salt tolerant genotypes under salt stress^16,27^.

There were several plant defense-related DEGs encoding NBS-LRR disease resistance proteins, NB-ARC containing proteins, terpene synthase, and 12-oxophytodienoate reductase. Due to proximity to a salt tolerance QTL, De Leon et al. (2016)^17^ suggested a NBS-LRR gene as a potential candidate enhancing salt tolerance in Pokkali. The pentatricopeptide repeat (PPR) proteins, which were crucial for adaptation under salinity^15,31^, overlapped with QTLs and carried large effect variants. Several dirigent genes, which have a role in passage of water and solutes into the vascular system due to its involvement in lignin synthesis^32^, were identified. These genes were upregulated in roots under salt stress in salt tolerant rice genotype SR86^13^. Similarly, the inclusion of glycosyl hydrolase family proteins in this list was obvious due to their response to biotic and abiotic stresses^33^.

The genes involved in signal transduction included members of OsGDSL (GDSL-like lipase/acylhydrolase) and jacalin-like lectin domain containing protein gene family^34–37^ and were differentially expressed in rice seedlings under salt stress^27,35,37^. Among kinases and phosphatases, most notable was receptor-like protein kinase 2 precursor which was represented by thirteen members of this gene family. Other genes included tyrosine protein kinase domain containing proteins, BRASSINOSTEROID INSENSITIVE 1-associated receptor kinase 1 precursor, and protein kinase domain containing proteins. A protein phosphatase 2C family protein OsPP2C8 (LOC_Os01g46760) was identified as a potential candidate gene for salt tolerance by associating sequence polymorphism with differential expression of genes present in the salt tolerance QTL region^38^. Although this gene was not detected in our study, we identified two other members (LOC_Os01g19130, LOC_Os02g13100) that carried SNPs and overlapped with QTLs.

Overall, the genetic complexity of salt tolerance is clearly evident from this study due to involvement of genes associated with multiple salt tolerance mechanisms; therefore, pyramiding of multiple beneficial alleles has been suggested to improve salinity tolerance in rice^39^. The candidate genes associated with salt tolerance were discovered by integrating the whole genome sequence analysis with QTL and gene expression data. However, this approach may not identify the genotype-specific salt tolerance genes and their variants because the salt tolerant donors vary widely in their response to salt stress due to the differences in genetic control of salt tolerance mechanisms. For example, different salt adaptation mechanisms operating in Pokkali and Nona Bokra^40^ could be the reason why no DEG was detected in the *Saltol* region. There are two most important outputs from this study: (1) the candidate genes with large impact variants in the coding as well as promoter regions represent some promising targets for validation in future; (2) the genomic resources of SNPs and InDels would be useful for genetic analysis and development of marker-assisted selection tools to improve salt tolerance in rice.

## Materials and methods

### Genome resequencing of rice genotypes with contrasting salinity response

Five rice genotypes with contrasting response to salt stress were used for this investigation. The salt tolerant genotypes, Pokkali and Nona Bokra, are *indica* landraces which are widely used in breeding program to enhance salt tolerance^3,4^ as well as in many QTL mapping studies^17–19,39,41^. Both genotypes respond differently to salt stress suggesting differences in salt tolerance mechanisms^40,42,43^. Among the salt sensitive genotypes, Bengal and Cocodrie are *japonica* cultivars developed at the Louisiana State University Agricultural Center^44,45^. Both varieties are highly sensitive to salt stress at the seedling stage^21^. IR64, a widely cultivated *indica* variety developed at the International Rice Research Institute, is sensitive to salt stress at the seedling stage^46^.

Genomic DNA was isolated from young leaves using Qiagen DNeasy kit (Qiagen Inc., Valencia, CA, USA). The quality and concentration of DNA in each sample were determined by Bioanalyzer 2100 (Agilent Technologies, Singapore) and Qubit 2.0 Fluorometer (Invitrogen Life Technologies, Eugene, Oregon), respectively. Libraries were made using Illumina TruSeq DNA sample preparation kit (Illumina, USA) and paired-end sequencing was done in an Illumina HiSeq 2000 at the Virginia Bioinformatics Institute, Blacksburg, VA. The filtering of raw sequence data was accomplished via an in-built standard Illumina pipeline.

The FASTQ files for all five genotypes were submitted to the sequence read archive (SRA) at the National Center for Biotechnology Information (NCBI) and the SRA accession numbers are PRJNA413821, PRJNA413822, PRJNA632686, and SRX272395.

### Mapping of whole genome sequences to the reference genome

The primer/adopter sequences and low-quality reads with Phred quality score < 30 were removed using the NGS QC Toolkit (v2.3.3; http://www.nipgr.res.in/ngsqctoolkit.html)^47^. Only high-quality filtered reads were used for mapping on the rice reference genome (MSU7 version; http://rice.plantbiology.msu.edu/index.shtml) using Burrows Wheeler Alignment (BWA) software (v0.7.12)^48^. The SAMtools (v1.1) was used to determine the reference genome coverage^49^. All downstream analyses were done using the uniquely mapped reads.

### Identification and annotation of variants

FreeBayes software (v0.9.21; https://github.com/ekg/freebayes) was employed to identify the SNPs and InDels using the following criteria: (a) the minimum variant frequency of ≥ 90%, (b) average quality of the SNP base ≥ 30, and (c) minimum read depth of 10. Additional filtering was done if there were three or more SNPs/InDels in any 10-bp window^50^. The distribution of DNA polymorphisms in different genomic regions was assessed by combining genome annotation information and positions of DNA polymorphisms. Above analyses including identification of synonymous/nonsynonymous SNPs, and large-effect SNPs/InDel were accomplished using single-nucleotide polymorphism effect predictor (SnpEff, v4.1 k)^51^ with default parameters. To identify the variants in the promoter regions, 2 kb upstream sequence of genes was used. All identified SNPs and InDels in the 5 rice genotypes compared to the reference genome were uploaded in a public depository Figshare (https://figshare.com/projects/Whole_genome_sequence_analysis_of_rice_genotypes_with_contrasting_response_to_salinity_stress_Insights_into_salt_tolerance/90053).

### Gene ontology analyses

BiNGO plug-in (version 2.44, https://www.psb.ugent.be/cbd/papers/BiNGO/Home.html) available in Cytoscape (version 3.2.2, http://www.cytoscape.org/) was used for gene ontology (GO) analysis at P-value of ≤ 0.05. For functional characterization, genes were assigned to eukaryotic orthologous group (KOG) using KOGnitor database of National Center for Biotechnology Information (NCBI). The GO enrichment analysis was done separately using genes with large-effect polymorphisms in all genes or promoter regions or the differentially expressed genes (DEGs), identified in all pair-wise comparisons as well as for those differentiating salt tolerant from salt sensitive groups (100% allelic variation between both groups).

### Mapping of SNPs/InDels on QTL regions

Since several QTL mapping studies were published by our laboratory using both Pokkali and Nona Bokra as salt tolerant donors^17–19,41^, the nonsynonymous SNPs and/or large effect variants (100% allelic variation) present in the QTL regions differentiating salt tolerant and salt sensitive groups were used to identify candidate genes associated with salt stress response. The start and end positions of QTLs in the MSU7 rice genome sequence were ascertained via BLASTN search. The nonsynonymous SNPs and/or large effect variants present within the QTL confidence intervals were identified based on overlapping of the genomic coordinates of QTLs in the rice genome annotation GFF file. The nonsynonymous SNPs and/or large effect variants present in the QTL confidence intervals were further mapped on the differentially expressed genes obtained between Pokkali and IR64 in response to salt stress in an earlier transcriptomic study^16^. This transcriptome study reported the differentially expressed genes (log2 fold change > 1 or < -1 with P-value ≤ 0.05) at the seedling stage under control and salinity stress using Tophat and Cufflinks pipeline^52^. After identification of variants in the QTL regions, the DEGs between tolerant and sensitive groups carrying these variants in CDS and promoter regions were analyzed for their functional relevance by GO analysis.

## Supplementary information

Supplementary Information 1.

Supplementary Information 2.

Supplementary Information 3.

Supplementary Information 3.

## References

[1] Munns R, Tester M (2008). Mechanisms of salinity tolerance. Ann. Rev. Plant Biol..

[2] Hasanuzzaman, M., Nahar, K., Alam, M.M., Bhowmik, P.C., Hossain, M.A., Rahman, M.M. *et al.* Potential use of halophytes to remediate saline soils. *J. Biomedicine Biotech.* **2014**, Article ID 589341 (2014). 10.1155/2014/589341 PMC4109415 25110683

[3] Akbar, M., Gunawardena, I.E. & Ponnamperuma, F.N. Breeding for soil stresses. In: *Progress in Rainfed Lowland Rice*. International Rice Research Institute, Manila (1986).

[4] Gregorio GB (2002). Progress in breeding for salinity tolerance and associated abiotic stresses in rice. Field Crops Res..

[5] Cushman JC, Bohnert HJ (2000). Genomic approaches to plant stress tolerance. Curr. Opin. Plant Biol..

[6] Nguyen KL, Grondin A, Courtois B, Gantet P (2018). Next-generation sequencing accelerates crop gene discovery. Trends Plant Sci..

[7] Huang X, Lu T, Han B (2013). Resequencing rice genomes: an emerging new era of rice genomics. Trends Genet..

[8] McNally KL (2009). Genome wide SNP variation reveals relationships among landraces and modern varieties of rice. Proc. Natl Acad. Sci. USA.

[9] Yamamoto T (2010). Fine definition of the pedigree haplotypes of closely related rice cultivars by means of genome-wide discovery of single-nucleotide polymorphisms. BMC Genom..

[10] Arai-Kichise Y (2011). Discovery of genome-wide DNA polymorphisms in a landrace cultivar of *japonica* rice by whole-genome sequencing. Plant Cell Physiol..

[11] Wang W (2018). Genomic variation in 3,010 diverse accessions of Asian cultivated rice. Nature.

[12] Han B, Huang X (2013). Sequencing-based genome-wide association study in rice. Curr. Opin. Plant Biol..

[13] Huang X (2012). A map of rice genome variation reveals the origin of cultivated rice. Nature.

[14] Jain M, Moharana KC, Shankar R, Kumari R, Garg R (2014). Genome-wide discovery of DNA polymorphisms in rice cultivars with contrasting drought and salinity stress response and their functional relevance. Plant Biotechnol. J..

[15] Chen R (2017). Whole genome sequencing and comparative transcriptome analysis of a novel seawater adapted, salt-resistant rice cultivar-sea rice 86. BMC Genom..

[16] Shankar R, Bhattacharjee A, Jain M (2016). Transcriptome analysis in different rice cultivars provides novel insights into desiccation and salinity stress responses. Sci. Rep..

[17] De Leon TB, Linscombe S, Subudhi PK (2016). Molecular dissection of seedling salinity tolerance in rice (*Oryza sativa* L.) using a high-density GBS-based SNP linkage map. Rice.

[18] Puram VRR, Ontoy J, Linscombe S, Subudhi PK (2017). Genetic dissection of seedling stage salinity tolerance in rice using introgression lines of a salt tolerant landrace Nona Bokra. J. Heredity.

[19] Puram VRR, Ontoy J, Subudhi PK (2018). Identification of QTLs for salt tolerance traits and prebreeding lines with enhanced salt tolerance using a salt tolerant donor ‘Nona Bokra’. Plant Mol. Biol. Rep..

[20] Chai C, Shankar R, Jain M, Subudhi PK (2018). Genome-wide discovery of DNA polymorphisms by whole genome sequencing differentiates weedy and cultivated rice. Sci. Rep..

[21] De Leon TB, Linscombe S, Gregorio G, Subudhi PK (2015). Genetic variation in Southern USA rice genotypes for seedling salinity tolerance. Front. Plant Sci..

[22] Wang L (2009). SNP deserts of Asian cultivated rice: genomic regions under domestication. J. Evol. Biol..

[23] Nagasaki H, Ebana K, Shibaya T, Yonemaru J, Yano M (2010). Core single-nucleotide polymorphisms-a tool for genetic analysis of the Japanese rice population. Breed. Sci..

[24] Smith JM, Haigh J (1974). The hitch-hiking effect of a favorable gene. Genet. Res..

[25] Wang S (2017). Integrated RNA sequencing and QTL mapping to identify candidate genes from *Oryza rufipogon* associated with salt tolerance at the seedling stage. Front. Plant Sci..

[26] Pandit A (2010). Combining QTL mapping and transcriptome profiling of bulked RILs for identification of functional polymorphism for salt tolerance genes in rice (*Oryza sativa* L.). Mol. Genet. Genom..

[27] Mansuri RM (2019). Dissecting molecular mechanisms underlying salt tolerance in rice: a comparative transcriptional profiling of the contrasting genotypes. Rice.

[28] Domingo C (2016). Physiological basis and transcriptional profiling of three salt-tolerant mutant lines of rice. Front. Plant Sci..

[29] Tiwari M, Sharma D, Singh M, Tripathi RD, Trivedi PK (2014). Expression of *OsMATE1* and *OsMATE2* alters development, stress responses and pathogen susceptibility in *Arabidopsis*. Sci. Rep..

[30] Xu J, Wang XY, Guo WZ (2015). The cytochrome P450 superfamily: Key players in plant development and defense. J. Integr. Agric..

[31] Jiang SC (2015). Crucial roles of the pentatricopeptide repeat protein SOAR1 in *Arabidopsis* response to drought, salt, and cold stresses. Plant Mol. Biol..

[32] Jin-long G (2012). A novel dirigent protein gene with highly stem-specific expression from sugarcane, response to drought, salt and oxidative stresses. Plant Cell Rep..

[33] Sharma R, Cao P, Jung KH, Sharma MK, Ronald PC (2013). Construction of a rice glycoside hydrolase phylogenomic database and identification of targets for biofuel research. Front. Plant Sci..

[34] Naranjo MA, Forment J, Roldan-Medina M, Serrano R, Vicente O (2006). Overexpression of *Arabidopsis thaliana* LTL1, a salt-induced gene encoding a GDSL-motif lipase, increases salt tolerance in yeast and transgenic plants. Plant Cell Environ..

[35] Jiang Y, Chen R, Dong J, Xu Z, Gao X (2012). Analysis of GDSL lipase (GLIP) family genes in rice (*Oryza sativa*). Plant Omics.

[36] Esch L, Schaffrath U (2017). An update on jacalin-like lectins and their role in plant defense. Int J. Mol. Sci..

[37] He X (2017). *OsJRL*, a rice jacalin-related mannose-binding lectin gene enhances *Escherichia coli* viability under high-salinity stress and improves salinity tolerance of rice. Plant Biol..

[38] Sun BR (2019). Genomic and transcriptomic analysis reveal molecular basis of salinity tolerance in a novel strong salt-tolerant rice landrace Changmaogu. Rice.

[39] Thomson MJ (2010). Characterizing the *Saltol* quantitative trait locus for salinity tolerance in rice. Rice.

[40] Moons A, Bauw G, Prinsen E, Van Montagu M, Van der Straeten D (1995). Molecular and physiological responses to abscisic acid and salts in roots of salt-sensitive and salt-tolerant *indica* rice varieties. Plant Physiol..

[41] De Leon TB, Linscombe S, Subudhi PK (2017). Identification and validation of QTLs for seedling salinity tolerance in introgression lines of a salt tolerant rice landrace ‘Pokkali’. PLoS ONE.

[42] Yeo AR, Yeo ME, Flowers SA, Flowers TJ (1990). Screening of rice (*Oryza sativa* L.) genotypes for physiological characters contributing to salinity resistance and their relationship to overall performance. Theor. Appl. Genet..

[43] Xie JH, Zapata-Arias FJ, Shen M, Afza R (2000). Salinity tolerant performance and genetic diversity of four rice varieties. Euphytica.

[44] Linscombe SD (1993). Registration of Bengal rice. Crop Sci..

[45] Linscombe SD (2000). Registration of ‘Cocodrie’ rice. Crop Sci..

[46] Kumari S (2009). Transcriptome map for seedling stage specific salinity stress response indicates a specific set of genes as candidate for saline tolerance in *Oryza sativa* L. Funct. Integr. Genom..

[47] Patel RK, Jain M (2012). NGS QC toolkit: a toolkit for quality control of next generation sequencing data. PLoS ONE.

[48] Li H, Durbin R (2009). Fast and accurate short-read alignment with Burrows–Wheeler transform. Bioinformatics.

[49] Li H (2009). The sequence alignment/map format and SAMtools. Bioinformatics.

[50] Jhanwar S (2012). Transcriptome sequencing of wild chickpea as a rich resource for marker development. Plant Biotechnol. J..

[51] Cingolani P (2012). A program for annotating and predicting the effects of single nucleotide polymorphisms, SnpEff: SNPs in the genome of *Drosophila melanogaster* strain w1118; iso-2; iso-3. Fly (Austin).

[52] Trapnell C (2012). Differential gene and transcript expression analysis of RNA-seq experiments with TopHat and Cufflinks. Nat. Protocol.

