# Supplementary material for: Whole genome sequence analysis of rice genotypes with contrasting response to salinity stress
Source: Sci Rep. 2020 Dec 4;10:21259. doi: 10.1038/s41598-020-78256-8 (PMC7719167; doi:10.1038/s41598-020-78256-8)
Supplement: Supplementary file 1 — Supplementary Information 1. [file 41598_2020_78256_MOESM1_ESM.pdf]

Supplementary Materials

Title: Whole genome sequence analysis of rice genotypes with contrasting response to salinity stress

Authors: Prasanta K. Subudhi[1], Rama Shankar[2], Mukesh Jain[2]

[1]School of Plant, Environmental, and Soil Sciences, Louisiana State University Agricultural Center, Baton Rouge, LA 70803, USA; [2]School of Computational and Integrative Sciences, Jawaharlal Nehru University, New Delhi 110067, India

This file includes Supplementary Tables S1-S5 and Supplementary Figures S1-S8.

Supplementary Tables S6, S7, and S8 were uploaded separately.

Supplementary Table S1. Distribution and frequency of SNPs (per 100 kb) between different rice cultivars on individual rice chromosome.

Supplementary Table S2. Distribution and frequency of InDels (per 100 kb) between different rice cultivars on individual rice chromosome.

Supplementary Table S3. List of SNPs and InDels detected in rice genotypes with respect to reference genome based on impact on mRNA and proteins.

Supplementary Table S4. List of SNPs and InDels detected in rice genotypes with respect to reference genome based on variant type and functional role.

Supplementary Table S5. Large-effect single-nucleotide polymorphisms (SNPs) and InDels detected in all six pair-wise combinations of rice genotypes.

Supplementary Table S6. List of salt tolerant QTLs identified in three mapping populations involving Pokkali and Nona Bokra as salt tolerant donors (De Leon et al. 2016; Puram et al. 2017, 2018)[17-19] to identify large effect variants in CDS and promoter regions of genes present in the QTL intervals (uploaded separately).

Supplementary Table S7. Differentially expressed genes harboring SNPs and/or InDels in promoter present in QTLs listed in Supplementary Table S6 (uploaded separately).

Supplementary Table S8. Differentially expressed genes harboring large effect SNPs and/or InDels in the promoter regions present in QTLs listed in Supplementary Table S6 (uploaded separately).

Supplementary Figure S1. Distribution and frequency of SNPs and InDels on individual rice chromosome detected in all pair-wise analysis of rice genotypes, Pokkali, Nona Bokra, Bengal, Cocodrie, and IR64. Number and frequency (per 100 kb) of SNPs (A) and InDels (B) detected on each rice chromosome are shown in the bar graphs.

Supplementary Figure S2. Number of SNPs and InDels between rice genotypes with contrasting response to salinity. Number of SNPs and Indels between salt sensitive (IR64, Bengal, and Cocodrie) and salt tolerant (Pokkali and Nona Bokra) rice cultivars are shown in the bar graphs.

Supplementary Figure S3. (A) Number of different substitution types in the identified SNPs detected in all pair-wise analysis of rice genotypes, Pokkali, Nona Bokra, Bengal, Cocodrie, and IR64. (B) Length distribution of InDels. Number of insertions and deletions (y-axis) of various lengths (x-axis, in bp) are shown in the bar graphs.

Supplementary Figure S4. Annotation of SNPs and InDels detected in all pair-wise analysis of rice genotypes, Pokkali, Nona Bokra, Bengal, Cocodrie, and IR64. (A) Distribution of SNPs and InDels in different genomic regions. (B) Distribution of SNPs (top) and InDels (bottom) in different genic regions. The number of synonymous (syn) and nonsynonymous (non-syn) SNPs and number of frameshift mutations are shown for SNPs and InDels, respectively.

Supplementary Figure S5. Functional categorization of genes identified with nonsynonymous/large-effect single-nucleotide polymorphisms (SNPs)/InDels. (A) Distribution of the eukaryotic orthologous group (KOG) classes in the genes identified with nonsynonymous SNPs in Bengal/Nona Bokra, Cocodrie/Nona Bokra, and IR64/Nona Bokra, (B) Distribution of the KOG classes in the genes identified with nonsynonymous SNPs in Bengal/Pokkali, Cocodrie/Pokkali, and IR64/Pokkali. Different KOG classes are listed on the right. P-value <0.05 is indicated by *.

Supplementary Figure S6. Gene ontology enrichment analysis for the genes harboring large effect/nonsynonymous SNPs and InDels (All SNPs/InDels detected in all pair-wise analysis of rice genotypes, Pokkali, Nona Bokra, Bengal, Cocodrie, and IR64).

Supplementary Figure S7. Gene ontology enrichment analysis for the genes harboring SNPs/InDels in the promoter regions (All SNPs/InDels detected in all pair-wise analysis of rice genotypes, Pokkali, Nona Bokra, Bengal, Cocodrie, and IR64).

Supplementary Figure S8. Gene ontology enrichment analysis for the differentially expressed genes (DEGs) harboring large effect SNPs/InDels and/or nonsynymous SNPs/InDels in the promoter regions (All SNPs/InDels detected in all pair-wise analysis of rice genotypes, Pokkali, Nona Bokra, Bengal, Cocodrie, and IR64 were included in this analysis).

Supplementary Table S1. Distribution and frequency of SNPs (per 100 kb) between different rice cultivars on individual rice chromosome.

Chr	Chr length (bp)	IR64/Pokkali		IR64/Nona Bokra		Bengal/Pokkali		Bengal/Nona Bokra		Cocodrie/Pokkali		Cocodrie/Nona Bokra	
		Count	Frequency	Count	Frequency	Count	Frequency	Count	Frequency	Count	Frequency	Count	Frequency
1	43270923	70987	164.1	63165	146.0	134354	310.5	149635	345.8	95019	219.6	102929	237.9
2	35937250	46650	129.8	58686	163.3	137742	383.3	131351	365.5	81750	227.5	80242	223.3
3	36413819	36282	99.6	47031	129.2	134359	369.0	138954	381.6	83002	227.9	85611	235.1
4	35502694	46433	130.8	39311	110.7	96234	271.1	107861	303.8	60714	171.0	70697	199.1
5	29958434	46060	153.7	50567	168.8	112318	374.9	109802	366.5	67603	225.7	66520	222.0
6	31248787	53029	169.7	51623	165.2	120597	385.9	113854	364.3	78115	250.0	73731	235.9
7	29697621	33090	111.4	32181	108.4	114496	385.5	113359	381.7	72735	244.9	72182	243.1
8	28443022	38737	136.2	64957	228.4	96643	339.8	96804	340.3	62644	220.2	61038	214.6
9	23012720	30454	132.3	24120	104.8	92155	400.5	89210	387.7	57032	247.8	55521	241.3
10	23207287	29266	126.1	33768	145.5	75077	323.5	84102	362.4	45904	197.8	52922	228.0
11	29021106	39541	136.2	46147	159.0	91703	316.0	92437	318.5	66010	227.5	64230	221.3
12	27531856	65792	239.0	37267	135.4	94840	344.5	86114	312.8	53606	194.7	48909	177.6
Sy[$]	633585	133	22.5	117	19.8	859	145.1	833	140.7	553	93.4	554	93.6
Un[$]	592136	409	64.6	357	56.3	457	72.1	395	62.3	259	40.9	237	37.4
Total	374471240	536,863	129.7	549,297	131.5	1,301,834	315.8	1,314,711	316.7	824,946	199.2	835,323	200.7

[$]Sy and Un represent the Syngenta and unanchored BAC pseudomolecules, respectively, as included in the MSU7 rice genome.

Supplementary Table S2. Distribution and frequency of InDels (per 100 kb) between different rice cultivars on individual rice chromosome.

Chr	Chromosome Length (bp)	IR64/Pokkali		IR64/Nona Bokra		Bengal/Pokkali		Bengal/Nona Bokra		Cocodrie/Pokkali		Cocodrie/Nona Bokra	
		Count	Frequency	Count	Frequency	Count	Frequency	Count	Frequency	Count	Frequency	Count	Frequency
1	43270923	6382	14.7	5709	13.2	11663	27.0	13288	30.7	7945	18.4	8801	20.3
2	35937250	4114	11.4	5430	15.1	11499	32.0	11433	31.8	6393	17.8	6464	18.0
3	36413819	3031	8.3	4118	11.3	11134	30.6	11968	32.9	6800	18.7	7307	20.1
4	35502694	3633	10.2	3131	8.8	7498	21.1	8587	24.2	4575	12.9	5355	15.1
5	29958434	3300	11.0	3724	12.4	8786	29.3	8818	29.4	4983	16.6	5070	16.9
6	31248787	4313	13.8	4437	14.2	9558	30.6	9278	29.7	5871	18.8	5669	18.1
7	29697621	2665	9.0	2819	9.5	9263	31.2	9432	31.8	5643	19.0	5661	19.1
8	28443022	3090	10.9	5149	18.1	7468	26.3	7940	27.9	4601	16.2	4854	17.1
9	23012720	2322	10.1	2047	8.9	7057	30.7	7159	31.1	4257	18.5	4246	18.5
10	23207287	2179	9.4	2602	11.2	5949	25.6	6802	29.3	3742	16.1	4248	18.3
11	29021106	3457	11.9	3915	13.5	7529	25.9	7941	27.4	4780	16.5	4690	16.2
12	27531856	5088	18.5	3372	12.2	7538	27.4	6897	25.1	4082	14.8	3906	14.2
Sy[$]	633585	9	1.5	8	1.4	53	9.0	60	10.1	30	5.1	29	4.9
Un[$]	592136	21	3.3	21	3.3	32	5.1	23	3.6	7	1.1	5	0.8
Total	374471240	43,604	10.3	46,482	10.9	105,027	25.1	109,626	26.1	63,709	15	66,305	15.5

[$]Sy and Un represent the Syngenta and unanchored BAC pseudomolecules, respectively, as included in the MSU7 rice genome.

Supplementary Table S3. List of SNPs and InDels detected in rice genotypes with respect to reference genome based on impact on mRNA and proteins.

Impact	SNPs	InDels
**High**	282 (0.23%)	95 (1.13%)
**Moderate**	4237 (3.52%)	13 (0.15%)
**Modifier**	112679 (93.51%)	8275 (98.31%)
**Low**	3296 (2.74%)	34 (0.40%)

Supplementary Table S4. List of SNPs and InDels detected in rice genotypes with respect to reference genome based on variant type and functional role.

	**SNPs**		**InDels**	
**Type of variants**	**Count**	**Percentage**	**Count**	**Percentage**
Missense variants	4,267	58.05%	0	0
Nonsense variants	197	2.68%	0	0
Silent variants	2,886	39.27%	0	0
splice_acceptor_variant	32	0.026%	1	0.012%
splice_donor_variant	23	0.019%	0	0
splice_region_variant	499	0.412%	38	0.449%
start_lost	10	0.008%	0	0
stop_gained	197	0.163%	0	0
stop_lost	20	0.017%	5	0.06%
stop_retained_variant	12	0.01%	0	0
conservative_inframe_deletion	0	0	5	0.059%
conservative_inframe_insertion	0	0	7	0.083%
disruptive_inframe_insertion	0	0	1	0.012%
frameshift_variant	0	0	94	1.111%

Supplementary Table S5. Large-effect single-nucleotide polymorphisms (SNPs) and InDels detected in all six pair-wise combinations of rice genotypes.

Type	Bengal vs Pokkali		IR64 vs Pokkali		Cocodrie vs Pokkali		Bengal vs Nona Bokra		IR64 vs Nona Bokra		Cocodrie vs Nona Bokra	
	InDels	SNPs	InDels	SNPs	InDels	SNPs	InDels	SNPs	InDels	SNPs	InDels	SNPs
Splice site acceptor	28	621	8	232	18	399	26	585	11	221	15	392
Splice site donor	32	515	13	206	16	297	28	500	9	199	19	293
Start lost	0	265	0	78	0	154	1	257	2	70	0	66
Stop lost	1	535	1	125	0	288	3	494	1	113	1	296
Stop gained	4	3476	4	1613	1	2163	3	3405	3	1613	1	2135
Frameshift	1800	0	842	0	955	0	1748	0	824	0	950	0
Total	1865	5412	868	2254	990	3301	1809	5241	850	2216	986	3182

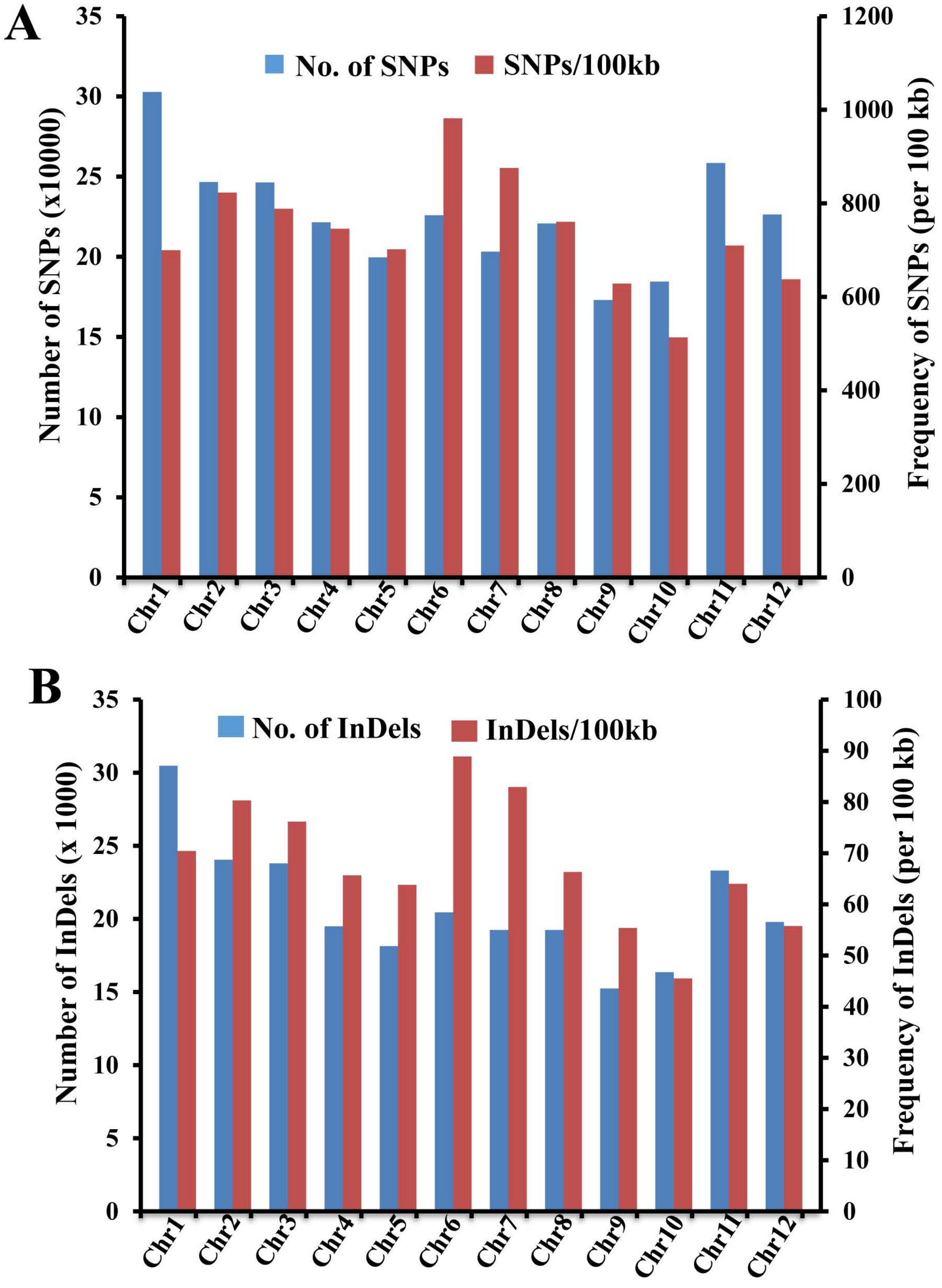

Supplementary Fig. S1 Distribution and frequency of SNPs and InDels on individual rice chromosome detected in all pair-wise analysis of rice genotypes, Pokkali, Nona Bokra, Bengal, Cocodrie, and IR64. Number and frequency (per 100 kb) of SNPs (A) and InDels (B) detected on each rice chromosome are shown in the bar graphs.

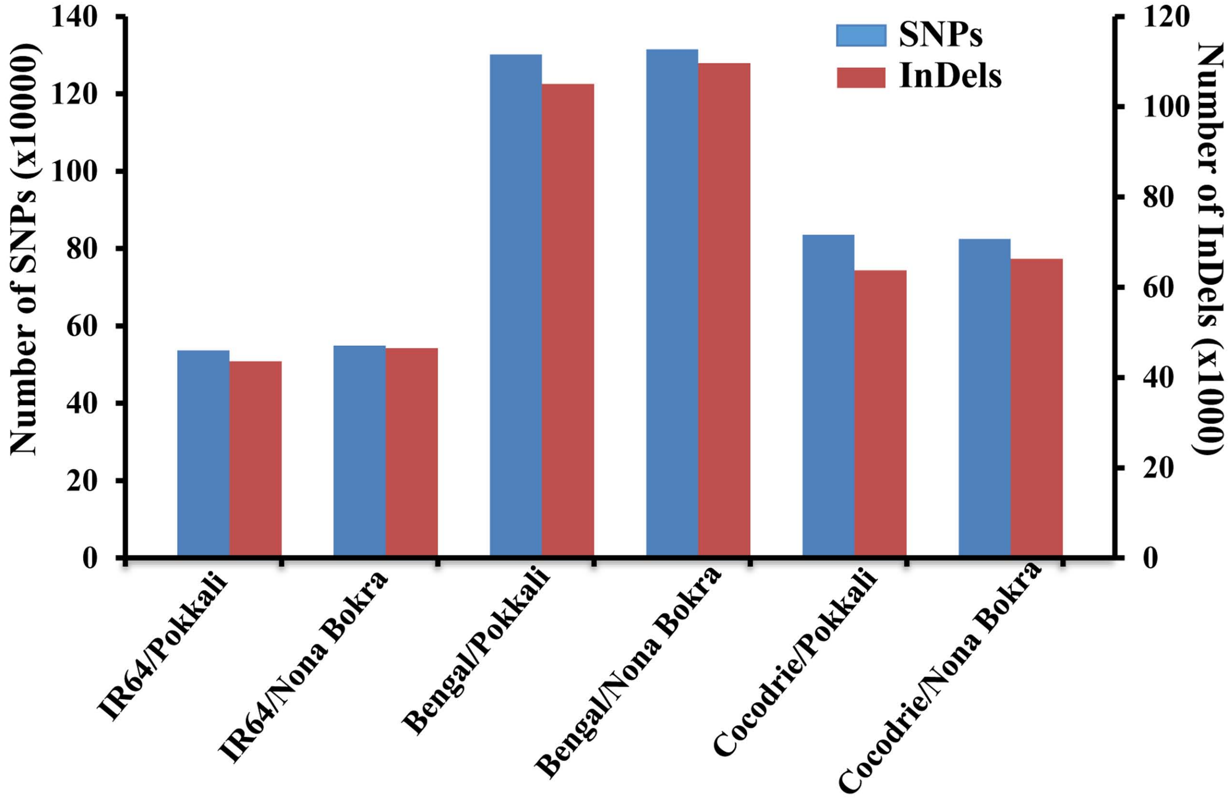

Supplementary Fig. S2 Number of SNPs and InDels between rice genotypes with contrasting response to salinity. Number of SNPs and Indels between salt sensitive (IR64, Bengal, and Cocodrie) and salt tolerant (Pokkali and Nona Bokra) rice cultivars are shown in the bar graphs.

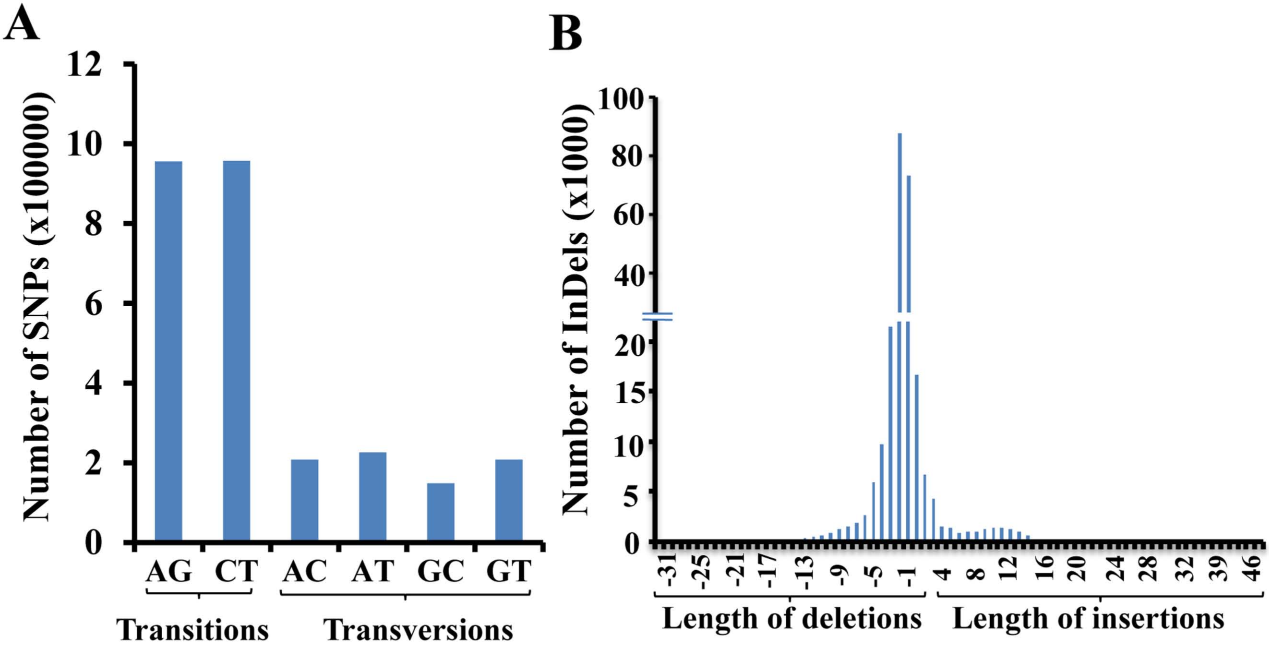

Supplementary Fig. S3 (A) Number of different substitution types in the identified SNPs detected in all pair-wise analysis of rice genotypes, Pokkali, Nona Bokra, Bengal, Cocodrie, and IR64. (B) Length distribution of InDels. Number of insertions and deletions (y-axis) of various lengths (x-axis, in bp) are shown in the bar graphs.

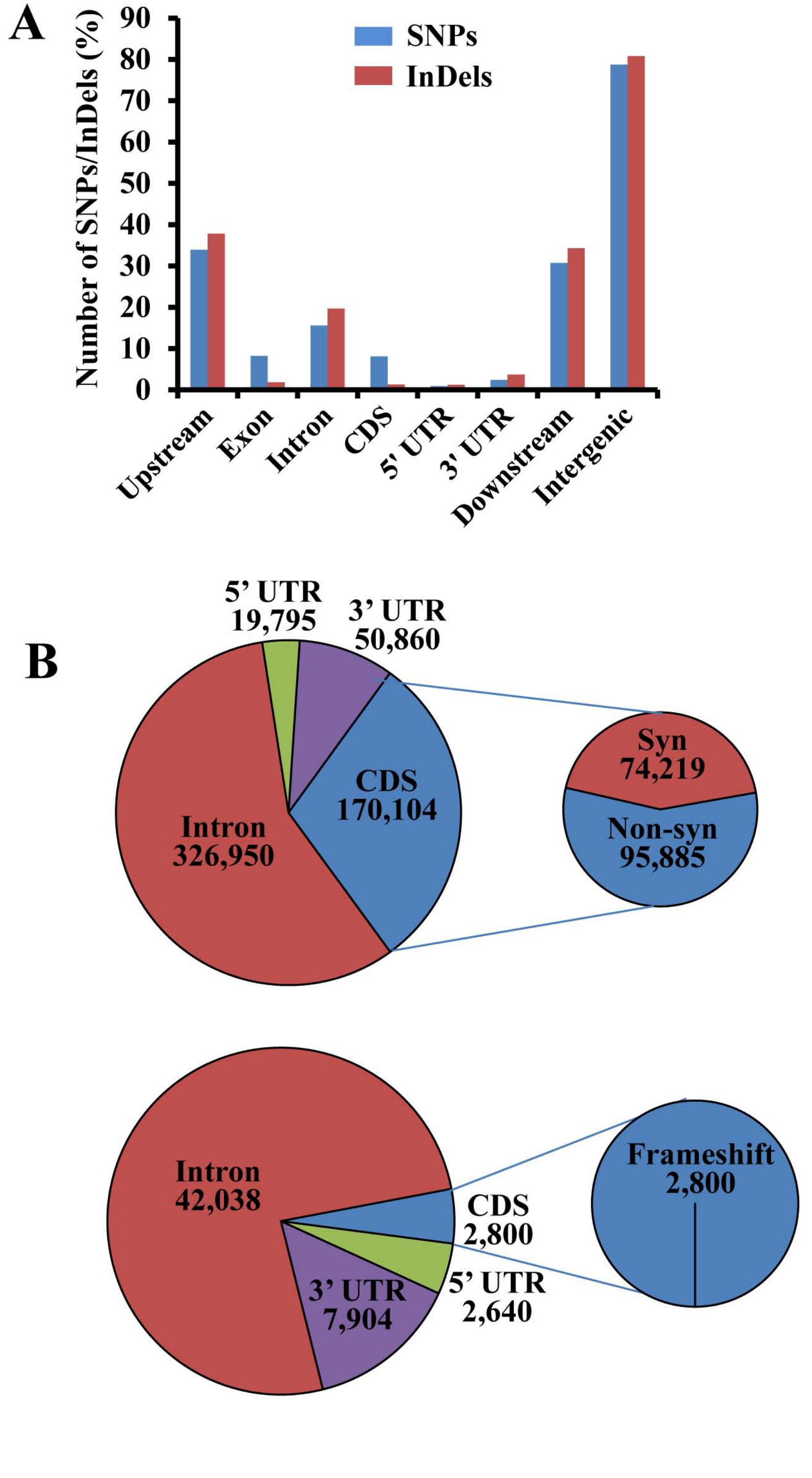

Supplementary Fig. S4 Annotation of SNPs and InDels detected in all pair-wise analysis of rice genotypes, Pokkali, Nona Bokra, Bengal, Cocodrie, and IR64. (A) Distribution of SNPs and InDels in different genomic regions. (B) Distribution of SNPs and InDels in different genic regions. The number of synonymous (syn) and non-synonymous (non-syn) SNPs and number of frameshift mutations are shown for SNPs and InDels, respectively.

A

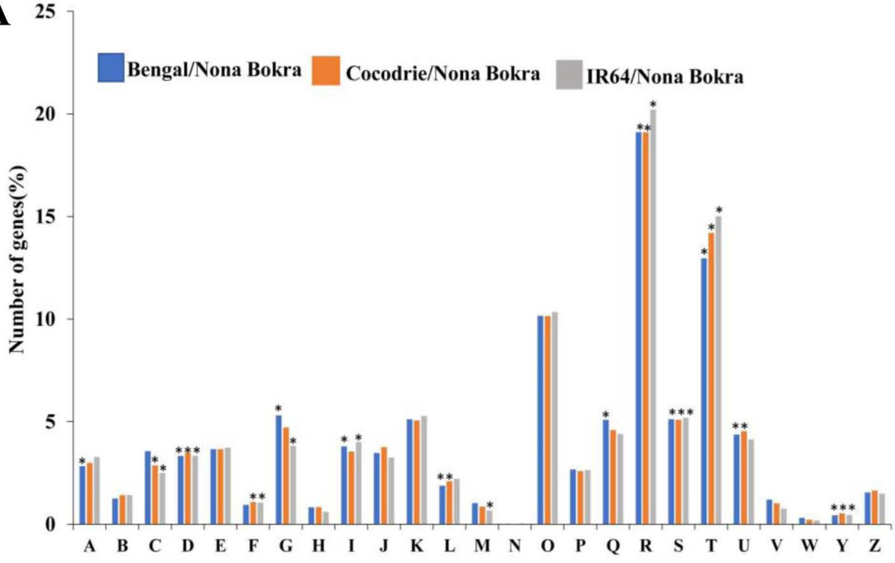

B

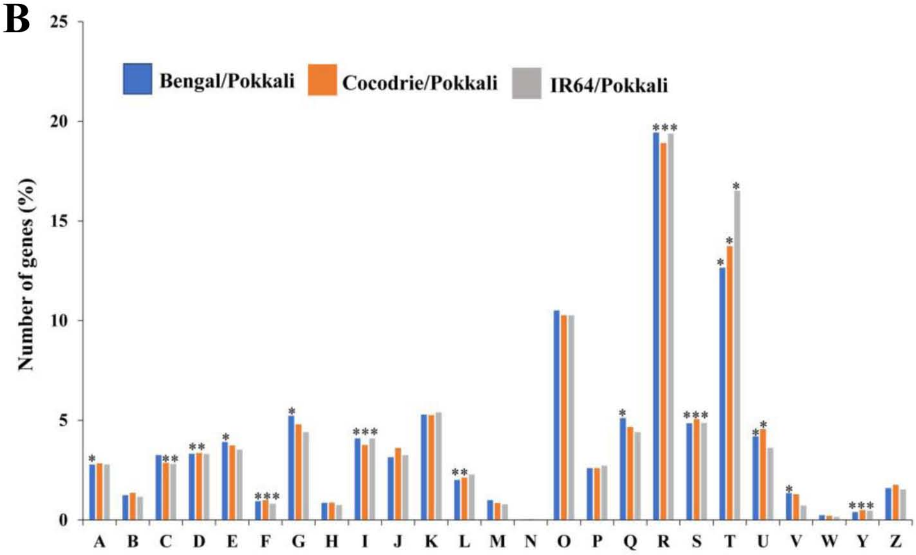

KOG ids	Description
J	Translation, ribosomal structure and biogenesis
A	RNA processing and modification
K	Transcription
L	Replication, recombination and repair
B	Chromatin structure and dynamics
D	Cell cycle control, cell division, chromosome partitioning
Y	Nuclear structure
V	Defense mechanisms
T	Signal transduction mechanisms
M	Cell wall/membrane/envelope biogenesis
N	Cell motility
Z	Cytoskeleton
W	Extracellular structures
U	Intracellular trafficking, secretion, and vesicular transport
O	Posttranslational modification, protein turnover, chaperones
C	Energy production and conversion
G	Carbohydrate transport and metabolism
E	Amino acid transport and metabolism
F	Nucleotide transport and metabolism
H	Coenzyme transport and metabolism
I	Lipid transport and metabolism
P	Inorganic ion transport and metabolism
Q	Secondary metabolites biosynthesis, transport and catabolism
R	General function prediction only
S	Function unknown

Supplementary Fig. S5 Functional categorization of genes identified with nonsynonymous/large-effect single-nucleotide polymorphisms (SNPs)/InDels. (A) Distribution of the eukaryotic orthologous group (KOG) classes in the genes identified with nonsynonymous SNPs in Bengal/Nona Bokra, Cocodrie/Nona Bokra, and IR64/Nona bokra, (B) Distribution of the KOG classes in the genes identified with nonsynonymous SNPs in Bengal/Pokkali, Cocodrie/Pokkali, and IR64/Pokkali. Different KOG classes are listed on the right. P-value <0.05 is indicated by *.

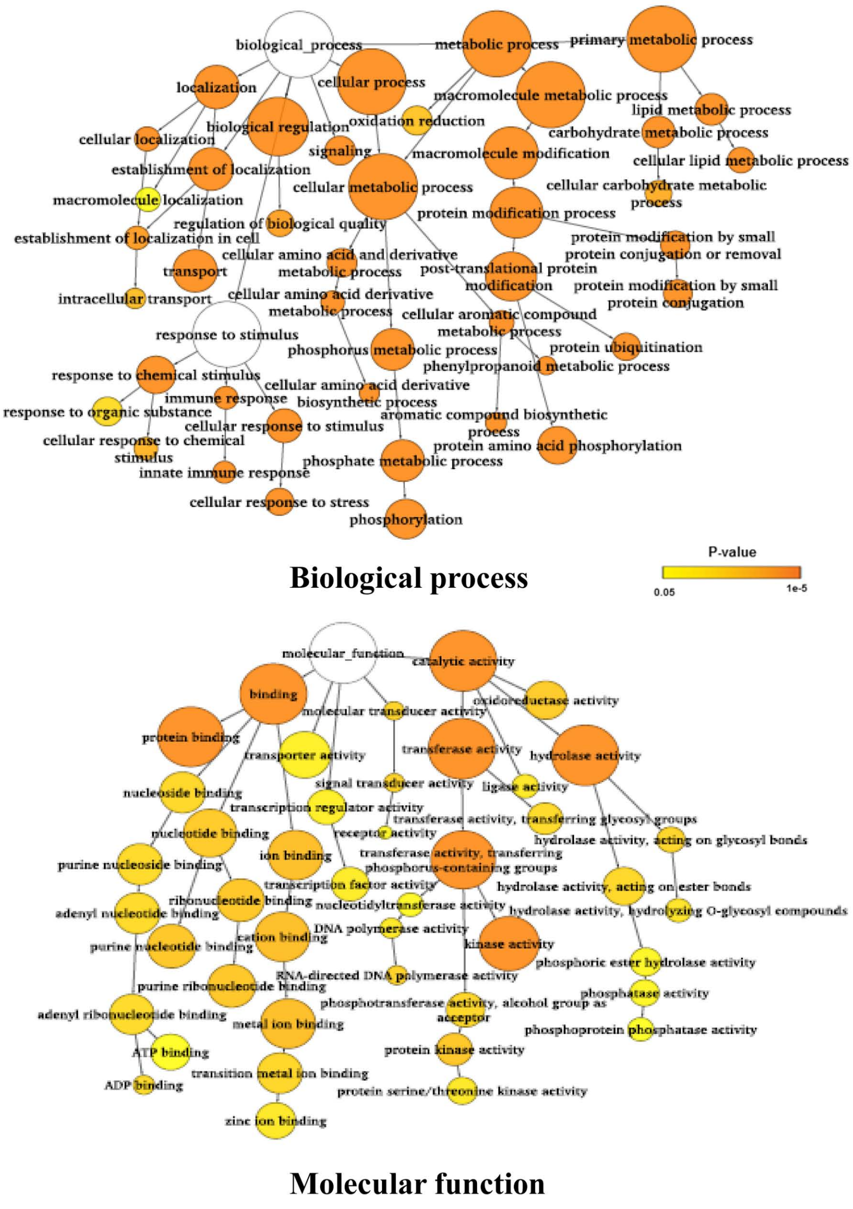

Supplementary Fig. S6 Gene ontology enrichment analysis for the genes harboring large effect/nonsynonymous SNPs and InDels (All SNPs/InDels detected in all pair-wise analysis of rice genotypes, Pokkali, Nona Bokra, Bengal, Cocodrie, and IR64).

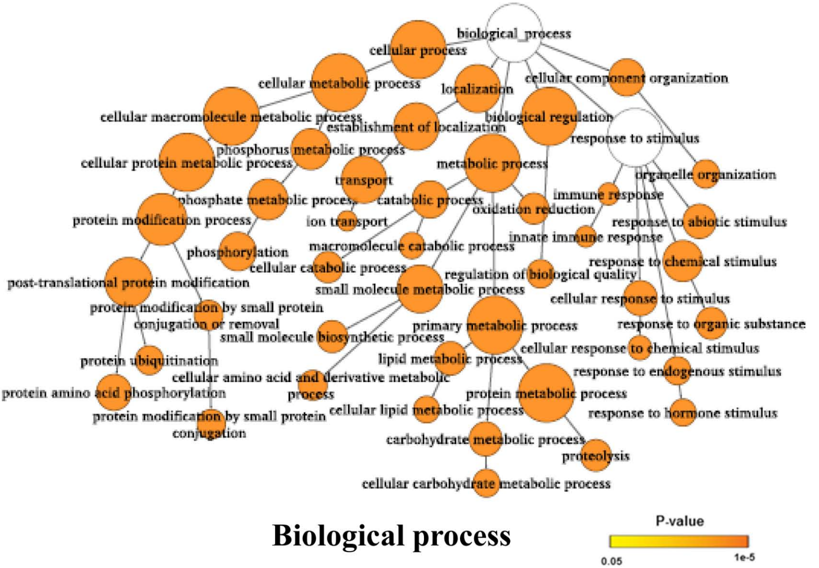

Biological process

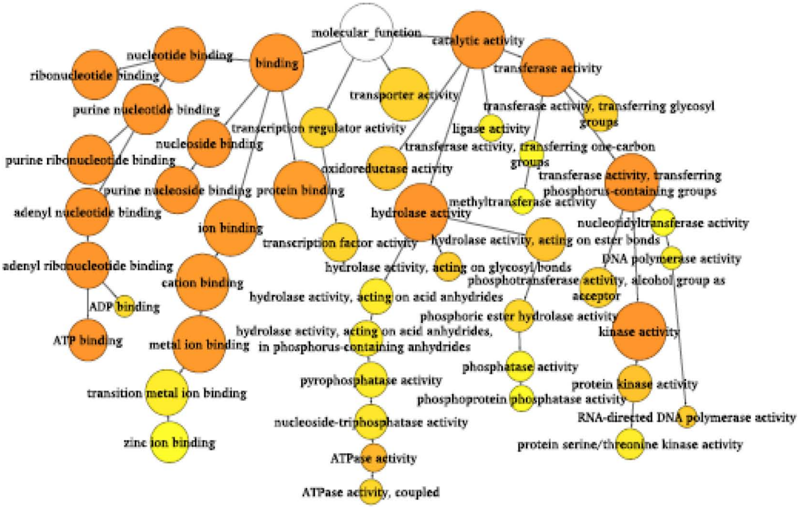

Molecular function

Supplementary Fig. S7 Gene ontology enrichment analysis for the genes harboring SNPs/InDels in promoter regions (All SNPs/InDels detected in all pair-wise analysis of rice genotypes, Pokkali, Nona Bokra, Bengal, Cocodrie, and IR64).

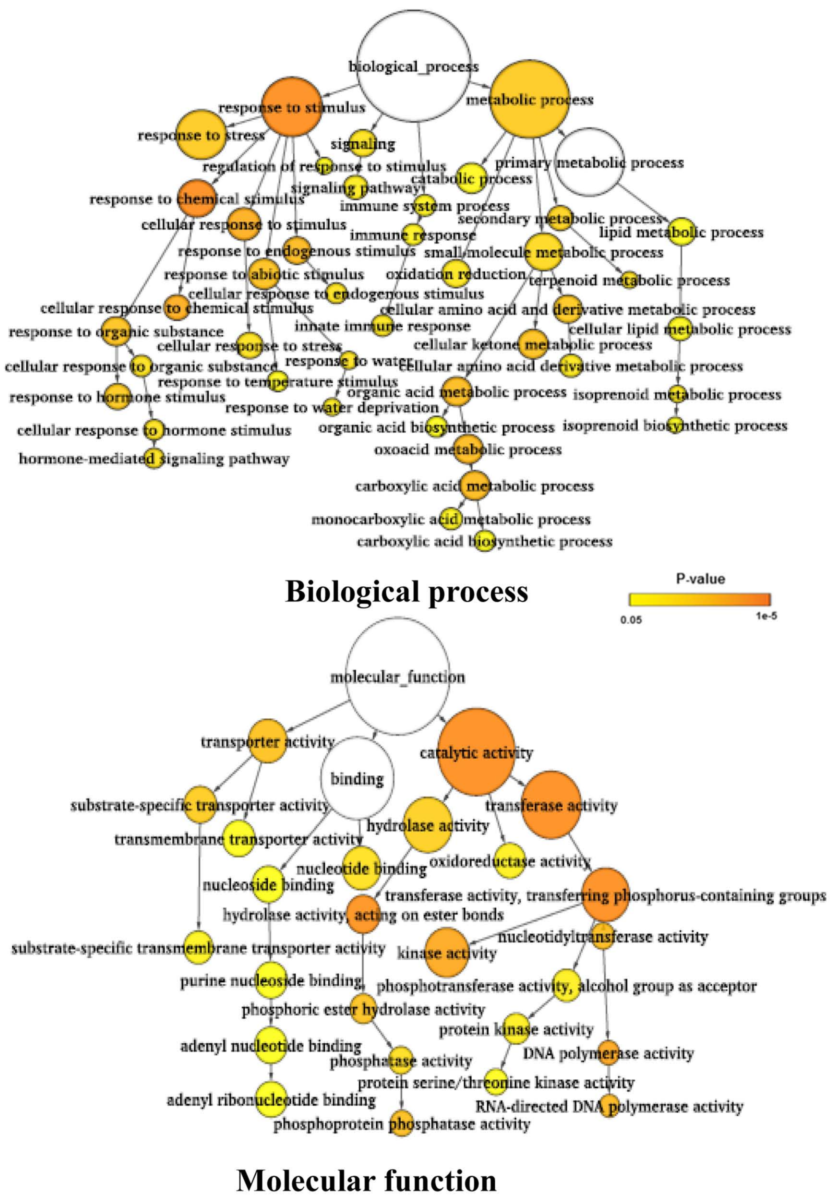

Supplementary Fig. S8 Gene ontology enrichment analysis for the differentially expressed genes (DEGs) harboring large effect SNPs/InDels and/or nonsynonymous SNPs/InDels in the promoter regions (All SNPs/InDels detected in all pair-wise analysis of rice genotypes, Pokkali, Nona Bokra, Bengal, Cocodrie, and IR64).
